# A Correlative SICM‐OPM Platform for Surface and Volumetric Imaging in Live Cells

**DOI:** 10.1002/advs.75222

**Published:** 2026-04-09

**Authors:** Wenzhi Hong, Ziwei Zhang, Ao Li, Ting Sun, Yunzhao Wu, Devkee M. Vadukul, Dylan Jones, Bing Li, Fengjie Liu, Francesco A. Aprile, Yuri Korchev, Julia Gorelik, David Klenerman, Andrew Shevchuk

**Affiliations:** ^1^ Faculty of Medicine Imperial College London London UK; ^2^ Department of Chemistry University of Cambridge Cambridge UK; ^3^ National Heart and Lung Institute ICTEM Imperial College London London UK; ^4^ Department of Chemistry Molecular Sciences Research Hub Imperial College London London UK; ^5^ Grantham Institute‐Climate Change and the Environment Department of Life Sciences Imperial College London London UK; ^6^ Institute of Chemical Biology Molecular Sciences Research Hub Imperial College London London UK; ^7^ Nano Life Science Institute (WPI‐NanoLSI) Kanazawa University Kanazawa Japan

## Abstract

Live‐cell imaging of cell surface topography and intracellular architecture is essential for understanding cellular function. However, conventional approaches often involve trade‐offs between resolution, invasiveness, and volumetric coverage. Here, we present an integrated Scanning Ion Conductance Microscope and single‐objective Oblique Plane Microscope (SICM‐OPM) system that enables simultaneous non‐contact topographical imaging and volumetric fluorescence imaging within the same live cell without sample translation. Beyond correlative live imaging, the platform supports nanomechanical mapping with tens‐of‐nanometers resolution, fluorescence‐guided localized molecular delivery via the SICM, and benefits from reduced photobleaching due to light‐sheet excitation. We demonstrate this platform's capabilities by visualizing imipramine‐induced transverse‐tubule (TT) remodeling in live cardiomyocytes, revealing pronounced detubulation of internal TT invaginations while surface TT opening characteristics remain largely preserved, and capturing high‐speed correlative volumetric images of clathrin‐mediated endocytosis in Cos‐7 cells. Additionally, we show precision delivery of fluorescent cargos, including dextrans and α‐synuclein, into mammalian cells and diatoms, alongside localized stiffness mapping to evaluate mechanical responses of mammalian cells. We believe this technique opens new avenues for correlative structural, functional, and biophysical studies in live cells, with broad relevance to cell biology, neurodegeneration, and mechanobiology.

## Graphical abstract

A multifunctional correlative imaging platform integrating Scanning Ion Conductance Microscopy (SICM) with Oblique Plane Microscopy (OPM) enables simultaneous surface topography, mechanical mapping, and 3D volumetric fluorescence imaging in live cells. This integrated system also supports targeted molecular delivery, offering new opportunities for studying cellular structure, dynamics, and local perturbations with high spatiotemporal precision.

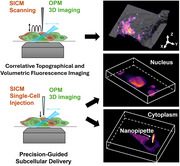

## 1 Introduction

Direct high‐resolution visualization of cell morphology and membrane dynamics is crucial for investigating key cellular processes, such as endocytosis, lipid raft formation, receptor clustering, transmembrane transport, membrane protein trafficking, and the passage of single nanoparticles or viruses—offering essential insights into cellular function in both health and disease. However, conventional imaging techniques face significant limitations: fluorescence microscopy is constrained by the diffraction limit, while super‐resolution methods either require high intensity laser powers, suitable fluorophores, many of which have considerable toxicity, or heavy image post‐processing [1]. Scanning electron microscopy (SEM) and correlated light and electron microscopy (CLEM) require fixation or introduce substantial disruption, making them unsuitable for live‐cell imaging [2]. Scanning ion conductance microscopy (SICM) has emerged as a powerful tool for non‐contact, nanometer‐resolution mapping of live‐cell surfaces [3]. By measuring ionic current changes through a nanopipette that scans over the cell surface, SICM generates quantitative topographical maps with nanometre precision, while avoiding mechanical interaction with the sample [4]. This makes SICM particularly suitable for imaging delicate cell types such as neurons, epithelial cells, cardiomyocytes, and even such complex tissue structures as the glomerulus [5, 6, 7, 8]. Furthermore, SICM has enabled detailed studies of membrane protrusions, ion channel distribution, and cellular mechanosensitivity, demonstrating its immense potential for probing cell membrane morphology and dynamics [9, 10, 11].

Despite the above strengths, SICM is fundamentally limited to two‐dimensional (2D) or quasi‐three‐dimensional (3D) representations of the cell surface and does not provide access to volumetric information inside the cell. Although fluorescence volumetric imaging can be achieved through confocal z‐stacking followed by 3D reconstruction, studies combining SICM with fluorescence confocal microscopy, e.g., scanning surface confocal microscopy (SSCM) and confocal laser scanning microscopy (CLSM), have demonstrated three‐dimensional structural correlation only on cell cytoplasmic membrane surfaces or thin biofilm systems [12, 13, 14]. These implementations have diffraction‐limited lateral and axial resolution, constrained by relatively slow volumetric acquisition, and induce excessive photobleaching. More recent developments include correlative combinations of SICM with stimulated emission depletion (STED) and super‐resolution optical fluctuation imaging (SOFI) that demonstrated super‐resolution in the *XY* plane, however, the axial resolution was demonstrated only for SOFI in chemically fixed cells and remained diffraction‐limited [15, 16]. Since the cell membrane operates in close coordination with internal cellular processes, and many surface dynamics events are influenced by or contribute to changes within the cell, understanding of such events requires simultaneous observation of membrane topography and intracellular activity [16]. One real‐time volumetric approach is to combine SICM with volumetric fluorescence imaging, such as light sheet fluorescence microscopy, which would offer the ability to monitor intracellular events. Nevertheless, integrating these two modalities exhibits several challenges. First, correlative SICM and fluorescence confocal microscopy achieves 3D surface imaging by moving the sample stage in 2D (X and Y plane), which would lead to a spatial mismatch between the two systems or significantly slow down acquisition speed due to the need for sequential, alternating movement [13]. Second, the physical setup of SICM typically places the nanopipette directly above the sample, leaving limited vertical space for integrating additional optical components, e.g., two orthogonal objectives commonly used in conventional light sheet fluorescence microscopy. To overcome these challenges, we developed a correlative volumetric live imaging platform based on a single objective fluorescence light sheet architecture, namely oblique plane microscopy (OPM) [17], which enables volumetric imaging without requiring orthogonal objectives or sample movement, and SICM that acquires topographical data by moving a nanopipette in 3D while keeping the sample stationary.

Unlike conventional volumetric imaging methods that require sample holder movement [18], OPM illuminates the specimen with a thin, oblique light sheet generated from a single objective positioned beneath the sample. By rapidly scanning the light sheet through the cell using a galvo mirror, OPM achieves optical sectioning without physically moving the sample. The resulting fluorescence images can be computationally stacked to reconstruct a 3D image of intracellular structures. Without sample movement and using a single objective for both sample illumination and fluorescence detection render OPM inherently compatible with the scanning mechanism and space requirements of SICM. Moreover, the light‐sheet‐based illumination confines excitation to a narrow plane within the cell, significantly reducing phototoxicity and photobleaching, making OPM particularly suited for long‐term live‐cell imaging and dynamic studies, such as intracellular dynamics, mitotic events, and calcium signaling [19, 20, 21, 22].

In this study, we present a novel implementation of a compact correlative SICM‐OPM system designed for comprehensive live‐cell imaging, utilizing the complementary strengths of both modalities. We describe the optical and mechanical integration of the system, along with dedicated pipelines for cross‐modality image registration and high‐resolution point‐based reconstruction. We demonstrate the versatility of the system across a range of live‐cell imaging tasks, including transverse‐tubule remodeling in live cardiomyocytes under drug treatment, visualization of well‐characterized clathrin‐mediated endocytosis in Cos‐7 cells, and precision‐guided nanopipette injection into neural cells and unicellular organisms such as diatoms. Importantly, our SICM‐OPM system is fully compatible with standard inverted microscope frames, facilitating its integration into conventional imaging workflows. Overall, we established a versatile SICM‐OPM platform for correlative imaging, targeted molecular perturbation, and local mechanical characterization across structurally and functionally diverse biological systems.

## 2 Methods

### 2.1 OPM System Description

**FIGURE 1:**
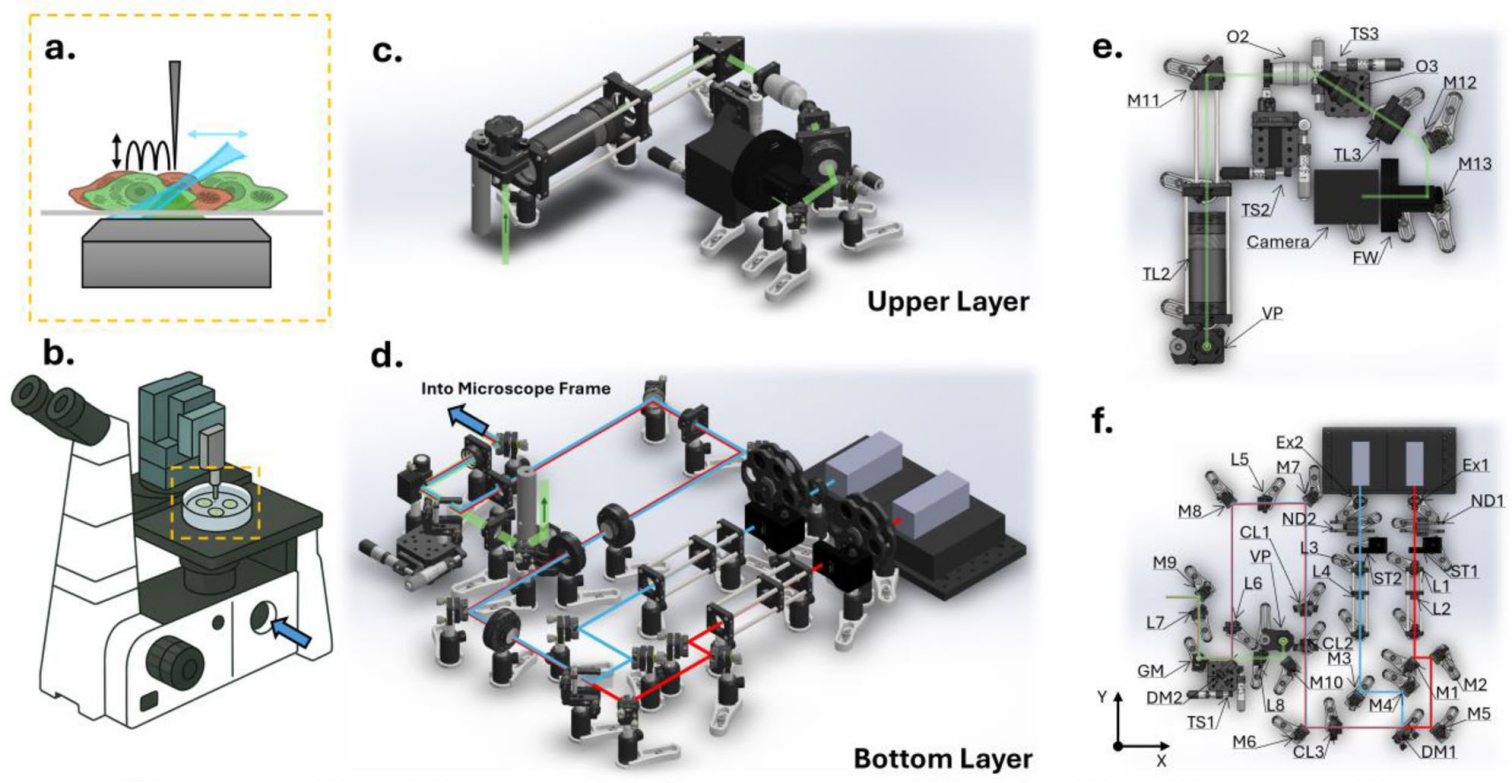
Layout of the SICM‐OPM system. The OPM system comprises two distinct optical layers. (a) Schematic illustration of the SICM‐OPM imaging principle. A hopping‐mode SICM nanopipette (black arrow) performs non‐contact nanoscale topographical imaging of the cell surface, while a tilted light sheet scans laterally (blue arrow) to enable fast volumetric fluorescence imaging. (b) Simplified diagram showing the SICM‐OPM system integrated into a standard inverted microscope frame. The SICM head is mounted above the sample stage, while the OPM detection and illumination optics are coupled into the side ports of the microscope frame. (c,d) 3D perspective views of the upper and bottom layers, respectively, illustrate the spatial arrangement of system components. (e) Top‐view schematic of the upper layer, showing the detection path. (f) Top‐view schematic of the bottom layer, detailing the common path and detection path. O, objective; L, lens; M, mirror; TL, tube lens; FW, filter wheel; DM, dichroic mirror; CL, cylindrical lens; VP, vertical periscope; TS, translation stage; Ex, excitation filter; GM, galvo mirror; ST, optical beam shutter; ND, neutral density filter.

In the OPM optical setup (see Figure 1), a high NA, 100 × silicone oil immersion objective (O1, NA 1.35, MRD73950, Nikon) is used both to provide an oblique, sheet‐like illumination within the sample and to collect the fluorescence emission. The collimated fluorescence emitted from O1 is then focused by a 200 mm Nikon tube lens (TL1), housed within a Nikon Eclipse TE2000‐U microscope frame. A pair of relay lenses (L7 and L8, AC254‐100‐A‐ML, Thorlabs) is positioned immediately after the microscope camera port to extend the optical path. In the common path between L7 and L8, a galvanometric mirror (GM, GVS201, Thorlabs) is placed at the focal plane of L7 to enable light‐sheet scanning during image acquisition. The fluorescence beam reflected from the galvo mirror then passes through a dichroic mirror (DM2, Di01‐R406/488/561/635‐25 × 36, Semrock), which redirects it upward via a vertical periscope system (VP, RS99/M, Thorlabs) to the upper detection layer. On the upper layer, the beam is collimated by a second tube lens (TL2), which comprises two commercial achromatic doublets (ACT508‐500‐A‐ML and ACT508‐1000‐A‐ML, Thorlabs), selected using the Doublet Selector [23]. An intermediate image is subsequently formed at the front focal plane of a secondary 40× air objective (O2, NA 0.95, CFI Plan Apochromat Lambda D, Nikon). This objective is mounted on a manual translation stage (LX30/M, Thorlabs), enabling precise pupil alignment. A glass‐tipped tertiary objective (O3, NA 1.0, AMS‐AGY v1.0, Calico Labs), also mounted on a manual translation stage (LX30/M, Thorlabs), is placed head‐to‐head with O2 at a 35° tilt. Finally, the image is focused onto a camera (sCMOS, PRIME‐BSI‐EXP, Photometrics) via a tube lens (TL3, TTL200‐A, Thorlabs) and a motorised emission filter wheel (FW, FE103/M, Thorlabs).

The illumination path is positioned on the bottom layer of the optical setup and begins with two laser sources (488 and 638 nm, 06‐MLD‐180/200, Cobolt, HÜBNER). Each laser is directed through an excitation filter (Ex1, FF01‐637/7‐25, Semrock; Ex2, MF475‐35, Thorlabs) followed by a dedicated neutral density filter wheel (ND1 and ND2, FW2AND, Thorlabs) and a beam shutter (ST1 and ST2; SH05R/M, Thorlabs). The beams are then passed through individual beam expanders, each comprising a pair of identical achromatic doublets (L1–L4, AC127‐025‐A‐ML, Thorlabs) with 1 × magnification. The 488 nm beam is reflected by a dichroic mirror (DM1, Di02‐R488‐25 × 36, Semrock), while the 638 nm beam is transmitted through DM1, allowing the two beams to combine and overlap downstream. After beam combination, the light passes through a cylindrical lens (CL1, ACY254‐100‐A, Thorlabs), which focuses the beam along one axis while leaving the orthogonal axis unaffected. An achromatic doublet (L5, AC254‐250‐A‐ML, Thorlabs) then collimates the focused axis and simultaneously begins to focus the previously unaffected direction. A second doublet (L6, AC254‐100‐A‐ML, Thorlabs), paired with L7, further relays the beam. The beam is then directed onto a galvanometric mirror (GM) via a dichroic mirror (DM2). The beam is ultimately focused through tube lens TL1 and objective O1 onto the sample plane, forming a light sheet in one axis while remaining collimated in the orthogonal direction (see Figure S1a). To maintain the light‐sheet thickness while extending its width, a cylindrical lens pair (CL2, ACY254‐200‐A, Thorlabs; CL3, ACY254‐050‐A, Thorlabs) is introduced between CL1 and the dichroic mirror DM1. This lens pair expands the beam size by a factor of 4 × in one direction, thereby increasing the light‐sheet width without compromising its thickness.

### 2.2 OPM Imaging Control

In the OPM system, lateral scanning of the light sheet was achieved using the galvo mirror (GM), controlled via a field programmable gate array (FPGA) board and a custom LabVIEW programme. For precise control of the galvo mirror angle, a voltage control port on the galvo mirror controller is connected to an analogue output channel (AO1) on the FPGA board. During system alignment, the mirror was driven with a fixed voltage of 1.5 V (see Figure S2b). During imaging mode, the galvo was scanned across 250 discrete steps with a voltage increment of 0.01 V per step, resulting in a lateral scan range of approximately 105 µm (see Figure S1b,c).

The LabVIEW program (see Figure S2a) also includes manual controls for the galvo mirror, enabling convenient sample monitoring. The scanning centre can be digitally adjusted within the program (see Figure S2b), avoiding the need for mechanical stage repositioning in some cases.

For synchronised galvo scanning and image acquisition, the camera was configured in the Edge Trigger mode via Micro‐Manager 2.0. In this mode, each frame was triggered by the rising edge of an external trigger signal generated through a digital output port (DIO1) on the FPGA. The camera's TRIG RDY output was connected to a digital input port (DIO2) on the FPGA, enabling the system to detect when the camera is ready to receive the next trigger. Once a high signal is detected on DIO2, the LabVIEW programme will send a rising edge via DIO1 to trigger the next frame.

An additional four digital output ports (DIO3‐DIO6) on the FPGA were used to control the emission filter wheel (FW) and two laser controllers, with two ports connected to the filter wheel's analogue inputs and the other two to the laser control inputs.

In single‐colour imaging mode, the emission filter wheel was fixed at a selected emission filter. In two‐colour imaging mode, the filters were automatically switched after each volumetric scan, as specified in the LabVIEW control sequence. Lasers were modulated in analogue mode via LabVIEW and were only turned on during image acquisition to minimize photobleaching.

### 2.3 SICM Description

SICM is a non‐contact scanning probe microscope that uses glass nanopipettes as imaging probes to produce topographical maps of sample surfaces submerged in liquid electrolyte, such as Phosphate‐Buffered Saline (PBS) which is widely used in biological research.

The SICM scan head was mounted on the frame of an inverted Nikon Eclipse TE2000‐U microscope (see Figure 1b). The coarse positioning of the SICM nanopipette was achieved by a PatchStar micromanipulator (Scientifica, UK), which provides 20 mm of travel in the *X*, *Y*, and *Z* axes.

Fine lateral (XY) scanning and positioning of the SICM pipette were performed using either an S‐316.10 Piezo Z/Tip/Tilt Scanner (Physik Instrumente, Germany), offering a scan range of 98 µm × 98 µm, or an S23.ZT1S‐C1 Tip/Tilt Scanner (CoreMorrow, China) with a 32 µm × 32 µm scan range. These Tip/Tilt scanners operate via three individually addressable piezo actuators arranged at the corners of an equilateral triangle, enabling precise control of both angular tilt and vertical displacement of the moving platform. To generate XY motion, the desired coordinates were converted into corresponding actuator voltages according to the *PZ 96E User Manual* (Physik Instrumente, Germany).

A custom Z‐axis piezo actuator was assembled from a stack of 12 PICMA piezo rings (PD050.30, Physik Instrumente, Germany) and mounted on the moving platform of the Tip/Tilt scanner. This Z actuator offers a travel range of 22 µm and a resonant frequency of 4 kHz, measured by an IDS3013 laser interferometer (Attocube, Germany). It was also equipped with a strain gauge sensor for closed‐loop control. All piezo actuators and sensors were controlled and read out using a Piezo Control System (ICAPPIC Ltd., UK). The microscope system was operated using custom HPICMScanner software and the ICAPPIC Universal Controller (ICAPPIC Ltd., UK).

SICM nanopipettes were fabricated from borosilicate glass capillaries (outer diameter 1.0 mm, inner diameter 0.5 mm; B‐100‐50‐10, World Precision Instruments) using a P‐2000 laser puller (Sutter Instruments, USA). Nanopipettes used for topographical and mechanical imaging were pulled using a two‐line program with the following parameters: Line 1: HEAT 350, FILAMENT 3, VELOCITY 30, DELAY 200, PULL 0 /Line 2: HEAT 340, FILAMENT 2, VELOCITY 27, DELAY 160, PULL 160. Note that these pulling parameters are specific to the individual instrument used and may require optimisation for different setups.

### 2.4 Imaging Processing Methods

The image processing in SICM‐OPM involves SICM height map reconstruction (flowchart see Figure S3a), OPM 3D image reconstruction [24], and correlative image registration (flowchart see Figure S3b). For SICM, the raw topological data encoded as a 2D RGB heat map is first converted to a grayscale height map, which is further transformed into a 3D binary volume with a uniform voxel size along the *X*, *Y*, and *Z* axes. Within this volume, the value of a voxel (*X*,  *Y*,  *Z*) is determined by:

$$\;I_{\left(X,\;Y,\;Z\right)\;}=\left\{\begin{array}{c} 255,\;\left(X=x,\;Y=y,Z=round\left(H\left(x,y\right)\right)\right),\\ 0,\;\left(X=x,\;Y=y,Z\neq round\left(H\left(x,y\right)\right)\right) \end{array}\right.$$

where *x*, *y*, and *H*(*x*, *y*) are the coordinates and height value of the corresponding pixel in the 2D height map, respectively. This transformation effectively reconstructs the SICM topography as a binary shell within the 3D volume.

In parallel, the OPM 3D image reconstruction is performed using a custom Python script [24]. The script first calls ImageJ to crop the region of interest (ROI) from the raw OPM image stack, ensuring that only the relevant portion of the data is processed. It then performs deskewing to reorient the tilted volume into its correct spatial alignment, making it suitable for quantitative analysis.

Next, the SICM binary shell within the 3D volume and the OPM 3D image volume are spatially aligned using a custom MATLAB‐based image registration pipeline. Due to the higher lateral resolution of SICM, the raw OPM volume is first rescaled in the X and Y dimensions to match the pixel resolution of the SICM data. A 2D grayscale version of the SICM height map is first used as the alignment reference. The rescaled OPM volume was loaded as a 3D array, and a candidate search centre, defined by a user‐input XY coordinate and a Z‐range, was specified to constrain the registration search region. The registration is then performed by scanning through each Z slice of the rescaled OPM stack within the defined range and testing local XY shifts (ΔX, ΔY) within a specified radius. For each potential alignment, a similarity metric (2D correlation coefficient generated by MATLAB *corr2* function) is calculated between the SICM height map and the OPM slice. The best‐matching position is identified based on the maximum similarity score. Once the optimal registration parameters were determined, the corresponding sub‐volume of the OPM stack was extracted and saved as the aligned OPM image stack. This aligned volume was then rescaled in the Z direction to restore isotropic voxel dimensions for downstream analysis and visualization.

To aid the registration process, a fluorescent spatial marker was introduced before each experiment by filling the SICM nanopipette with fluorescently labeled dextran (D22914, Thermo Fisher). The labeled pipette was imaged using the OPM, providing a visible reference point in the fluorescence volume that corresponded to the SICM scanning center. This marker ensured that the initial search region could be centred near the true SICM imaging location, improving both accuracy and efficiency.

### 2.5 High‐Resolution Point‐Based (HRPB) Reconstruction of Transverse‐Tubules From OPM Fluorescence Images

To improve the visualization of transverse‐tubule (TT) structures in cardiomyocytes, a custom MATLAB‐based image reconstruction pipeline was developed to generate high‐resolution point‐based (HRPB) image stacks from raw OPM fluorescence images.

Each frame of the OPM stack was first converted to double precision and processed using a Laplacian of Gaussian (LoG) filter to enhance and detect point‐like features. The LoG filter parameters were chosen based on the measured system point spread function (0.46 µm FWHM). A fixed intensity threshold was applied to suppress background fluorescence, and local maxima in the filtered image were identified as candidate TT localizations.

To isolate meaningful structures and eliminate background artefacts, a binary cell mask was generated based on intensity thresholding. Small objects and features near image borders were removed, and localization candidates falling outside the cell mask were excluded from further analysis.

For each accepted localization, the local signal intensity was quantified using a circular region of interest. Alternatively, an option was provided to assign a uniform intensity to all localizations to better visualize the geometric organization of TTs, independent of fluorescence variation.

The coordinates of detected localizations were then used to construct a high‐resolution image by upsampling the original frame by a factor matched to the pixel resolution of the corresponding SICM data. Each localization was rendered as a Gaussian spot, using a kernel whose width was scaled to reflect the measured system PSF in the upsampled space, see Section 3.1. A high‐resolution image was generated for each frame by summing the contributions of all localizations (see Figure S4).

While the current implementation does not perform super‐resolution reconstruction beyond the diffraction limit, it enhances the spatial representation of subcellular features by resampling and re‐rendering localized signals on a finer spatial grid. This approach improves structural clarity and supports quantitative analysis of quasi‐periodic or punctate structures, such as TT openings, without requiring hardware‐based super‐resolution methods. For continuous organelles (e.g., endoplasmic reticulum or mitochondria), standard diffraction‐limited OPM volumetric imaging remains applicable. Notably, the underlying framework allows for future extension toward true super‐resolution reconstruction by adapting the detection kernel to smaller effective PSFs corresponding to finer imaging modalities.

### 2.6 SICM Injection

Fluorescently labeled cargos were delivered into target cells, including *Coscinodiscus radiatus* diatoms and neuroblastoma cells, using a localized electroporation approach enabled by SICM nanopipette injection [25]. The nanopipette, pre‐loaded with the appropriate cargo solution, was precisely positioned vertically above the target cell surface. The pipette was advanced toward the cell surface until a 0.5% drop in ion current was detected relative to the baseline (i.e., when the pipette was distant from the surface), indicating proximity to the cell. The pipette was then lowered an additional 2 µm to ensure membrane contact.

A sequence of 20 electroporation pulses was delivered by applying a voltage protocol to the reference electrode in the bath:−10 V for 50 ms, +10 V for 5 ms, and −0.1 V for 2 ms per episode. Nanopipettes for this injection experiment were fabricated using a P‐2000 laser puller (Sutter Instruments, USA) with the following two‐line program: Line 1: HEAT 280, FILAMENT 3, VELOCITY 20, DELAY 150, PULL 0 /Line 2: HEAT 300, FILAMENT 4, VELOCITY 15, DELAY 120, PULL 100. Note: Pulling parameters are specific to the individual puller used and may require optimisation for other instruments.

### 2.7 Biological Sample Preparation Methods

#### 2.7.1 Diatom Cultivation

The diatom *Coscinodiscus radiatus* (CCMP312) was obtained from the Provasoli‐Guillard National Center for Marine Algae and Microbiota (NCMA, USA). The culture was grown in artificial seawater medium [26] and in a controlled environmental growth room at 23°C with an illumination of 50 µmol·m^−2^·s^−1^ (12 h light / 12 h dark) at Silwood Park campus of Imperial College London. The seawater medium was prepared using aseptic techniques, and laboratory materials were acid‐cleaned. Chemicals of ACS grade or higher purity were used, and the artificial seawater was 0.2 µm filtered (polycarbonate filters, Merck Millipore Ltd.) and kept at 4°C in the dark before use. Fresh batches were inoculated in a microbiological safety cabinet by transferring 5 mL of culture to 35 mL of media in a new 40 mL culture flask fortnightly.

#### 2.7.2 Adult Rat Cardiomyocyte Preparation

Adult rat ventricular cardiomyocytes (ARVMs) were isolated from male adult Sprague‐Dawley (SD) rats (150–250 g) using a Langendorff perfusion system. Following isolation, approximately 10 000 cardiomyocytes were seeded onto 35 mm glass‐bottom dishes (P35G‐1.5‐14‐C, MatTek) pre‐coated with laminin.

Cells were initially incubated in Minimum Essential Medium (MEM, Cat.#31095029, Gibco) supplemented with 10% fetal bovine serum (FBS, Thermo Fisher) and 1% Antibiotic‐Antimycotic Solution (Cat.#A5955, Sigma) for 1 h at 37°C in a humidified atmosphere with 5% CO_2_. The medium was replaced with serum‐free MEM for subsequent treatments.

To induce TT disruption, cells were treated with 300 µm of imipramine hydrochloride (Cat.#7841, Tocris Bioscience) diluted in serum‐free MEM. The ARVMs were incubated with the imipramine solution for 15 min at 37°C, 5% CO_2_.

For membrane staining, a Di‐8‐ANEPPS working solution was prepared by diluting a 2 mm stock to a final concentration of 20 µm in a high‐potassium buffer (120 mm K‐gluconate, 25 mm KCl, 2 mm MgCl_2_, 1 mm CaCl_2_, 2 mm EGTA, 10 mm HEPES, 10 mm glucose, adjusted to pH *7.4*). The solution was sonicated in a water bath at 55°C for 5 min.

Cells were washed twice with high‐potassium buffer and stained with the Di‐8‐ANEPPS solution for 5 min at room temperature (RT) in the dark, followed by high‐potassium buffer washing twice and immediate imaging.

All animal experiments were carried out in accordance with the United Kingdom Home Office Animals (Scientific Procedures) Act 1986, Amendment Regulations 2012, incorporating the EU Directive 2010/63/EU.

#### 2.7.3 SH‐SY5Y Human Neuroblastoma Cell Line

SH‐SY5Y human neuroblastoma cells were cultured at 37°C and 5% carbon dioxide in Roswell Park Memorial Institute medium (RPMI 1640, Thermo Fisher) with 10% fetal bovine serum (FBS, Thermo Fisher). Trypsin‐EDTA (0.25%, Thermo Fisher) was used to detach cells from the culture plate, and culture medium was refreshed every 2–3 days. SH‐SY5Y cells were harvested at ∼70% confluency and subsequently plated on glass‐bottomed dishes (P35G‐1.5‐14‐C, MatTek). MatTek dishes were coated with Poly‐D‐lysine (0.1 mg/mL) and collagen from rat (0.1 mg/mL) and incubated at RT for 1 h, prior to washing three times with Dulbecco's phosphate‐buffered saline (DPBS) and subsequent cell seeding.

#### 2.7.4 α‐synuclein Monomers Tagged With Alexa Fluor 488

α‐synuclein carrying the A140C mutation (A140C α‐syn) was purified using a previously reported protocol [27]. Briefly, the protein was overexpressed in *E. coli* BL21(DE3) overnight with 1 mm IPTG, followed by cell lysis, streptomycin sulfate precipitation of nucleic acids, and ammonium sulfate precipitation of proteins. α‐Syn was further purified by ion‐exchange chromatography on a HiPrep Q HP 16/10 column (Cytiva) using a 0–1 m NaCl linear gradient, followed by size‐exclusion chromatography on a HiLoad 16/600 Superdex 75 pg column (GE Healthcare) in standard PBS. All steps were performed in the presence of 0.5 mm DTT.

Labeled α‐syn monomers were obtained by conjugation of A140C α‐syn with Alexa Fluor 488 C5‐maleimide (Thermo Fisher). A140C α‐syn was buffer‐exchanged using Zeba Spin Desalting Columns (7K molecular weight cutoff) (Thermo Fisher) to remove the DTT from the storage solution. The reaction was carried out according to the manufacturer's instructions.

#### 2.7.5 Cos‐7 Cell Line and Clc‐GFP Transient Transfection

Green Monkey Cos‐7 cells were maintained at 37°C in 5% CO2 using DMEM (Life Technologies) containing 5% (volume/volume) FCS, seeded at 1 × 106 cells per T25 flask. For SICM‐OPM imaging, cells were plated for 24 h on glass‐bottom Mattek dishes (P35G‐1.0‐14‐C, Mattek Corp., USA) and then transfected with Clc‐GFP as follows. Cells were washed before transfection with PBS, and complexes of Lipofectamine (Life Technologies) and plasmid DNA at a ratio of 1 µL to 1 µg were added in Opti‐MEM (Life Technologies) without FCS to the cells. After 2 h, the media was replaced with DME containing 5% FCS. The plasmid DNA used in the experiments was pCi (Promega) containing clathrin light chain‐GFP (provided by L.E. Greene, Laboratory of Cell Biology, National Heart, Lung, and Blood Institute, National Institutes of Health, Bethesda, MD; Wu et al., 2001). Cells were imaged 24 h past transfection in HBSS with calcium and magnesium without phenol red (Gibco).

## 3 Results

### 3.1 SICM‐OPM Design

The SICM‐OPM system was constructed on an inverted Nikon Eclipse TE2000‐U microscope frame, enabling mechanical integration of the SICM scan head with the optical components of the oblique plane microscopy (OPM) module (Figure 1). The SICM scan head was mounted directly onto the microscope frame, providing stable positioning of the nanopipette above the sample while maintaining unobstructed access to the optical path.

A critical requirement in the OPM alignment was ensuring that the back focal plane of the primary objective (O1), the galvo mirror (GM), and the back focal plane of the remote objective (O2) were optically conjugated [28, 29]. This conjugation guarantees that angular deflection of the galvo mirror results in pure lateral translation of the light sheet at the sample plane, without altering beam position at intermediate conjugate planes. As a result, volumetric scanning can be performed without introducing lateral image distortion or scan‐dependent aberrations.

Beam shaping was achieved using a sequence of cylindrical lenses (CL1, CL2, and CL3), all aligned along the same axis of curvature. This configuration ensures that their focal power is applied consistently along a single direction, producing uniform beam shaping throughout the optical path. The coordinated action of these cylindrical elements generates a thin, elongated illumination profile suitable for light‐sheet formation.

Together, this optical configuration produces a thin, obliquely oriented light sheet that can be rapidly scanned through the cell volume via the galvo mirror while the sample remains stationary. This architecture enables fast volumetric fluorescence imaging that is fully compatible with SICM operation. The measured light‐sheet thickness at the sample plane was approximately 1.2 µm, with a full‐width‐at‐half‐maximum (FWHM) width of 82 µm.

**FIGURE 2:**
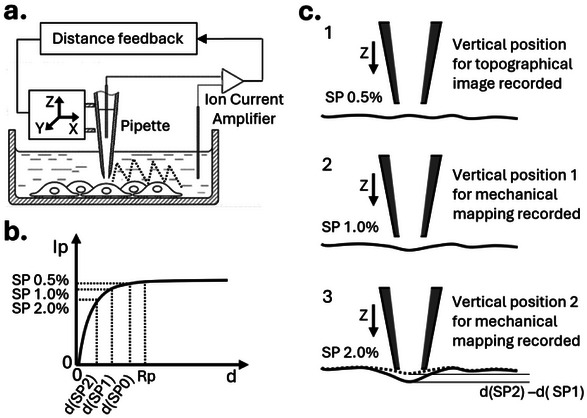
SICM principles of imaging and stiffness measurements. (a) Schematic diagram (not to scale) shows SICM ion current measurement electrodes connections, a nanopipette mounted on a 3D piezo scanner, and immersed in a petri dish with cell culture submerged in physiological buffer medium. The dashed line shows the pipette scan trajectory in Hoping Probe mode. (b) Approach characteristic plot showing the relationship between set point expressed as a percentage of ion current versus pipette to sample surface separation distance. Ion current is normalized to the maximum current registered when the pipette is further than one radius of opening away from the surface. Distance is expressed in pipette opening radius. (c) Progressive deformation of the surface with increased set point (SP).

The SICM module was integrated directly above the sample plane (see Figure 1b), allowing non‐contact topographical and mechanical measurements to be performed concurrently with optical imaging. SICM nanopipettes are filled with electrolyte, accommodate a measurement electrode, and mounted on a 3D piezo manipulator that performs scanning (see Figure 2a). When a bias voltage potential, typically 200 mV, is applied between the measurement electrode inside the pipette and a reference (ground) electrode immersed in a petri dish, an ion current is generated. If the pipette tip approaches a surface that is not permeable to ions at a distance closer than one radius of its opening (Rp), the current through the pipette (Ip) starts dropping (see Figure 2b) [30]. When Ip drops below a specified Set Point (SP), typically by 0.5% from the maximum Ip value registered in the bulk, the vertical position of the piezo is stored in a topographical surface map for the current XY coordinate. Then the piezo withdraws the pipette, positions to a new XY coordinate, and repeats the approach to make a new height measurement. Such an imaging mode is called “hopping” [3]. For mechanical mapping at each imaging XY coordinate, the pipette is moved further down until Ip drops by 1% (SP1) and then by 2% (SP2), as shown in Figure 2b. This results in local surface deformation because a positive bias voltage applied to the measurement electrode inside the SICM pipette generates outward flow that converts into pressure when the gap between the pipette and the surface becomes smaller (see Figure 2c) [31]. The deformation, measured as the difference in vertical positions corresponding to SP1 and SP2 is then converted to Young's modulus as previously published [32].

### 3.2 Optical System Characterisation With Fluorescent Beads

We first estimated the theoretical resolutions of the system based on the following parameters: an effective numerical aperture NA (35° tilted configuration, see Figure 1e) of 1.33, emission wavelength of 525 nm, bead diameter of 100 nm, camera binned pixel size of 13 µm, and an effective lateral magnification of 56 ×. Assuming that these contributions to the point spread function (PSF) can be approximated as independent Gaussian distributions, the theoretical lateral FWHM was estimated to be 0.32 µm, and the theoretical axial FWHM was 0.74 µm.

**FIGURE 3:**
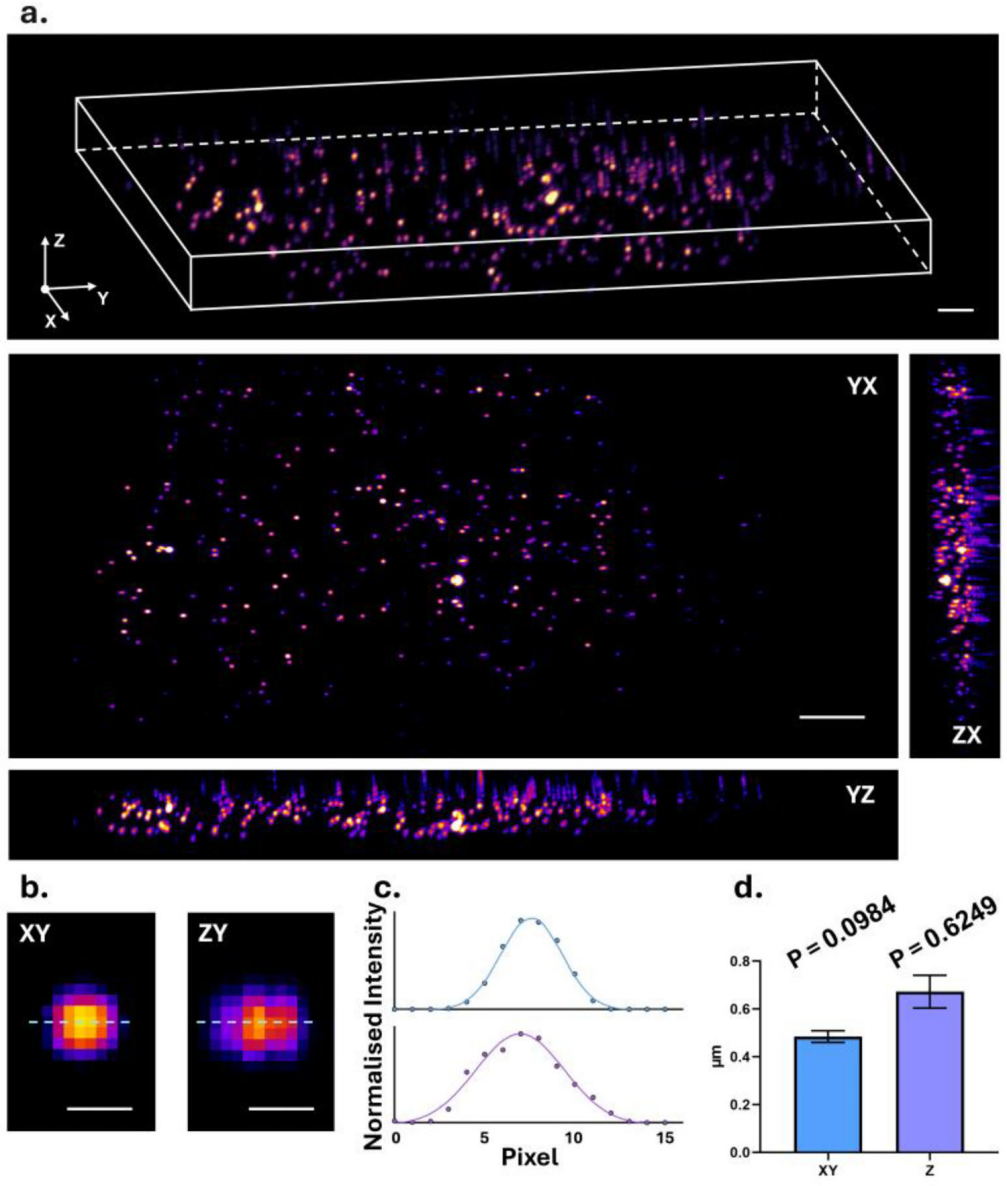
Optical characterisation of the OPM system using 100 nm diameter fluorescent beads. (a) 3D perspective view and maximum intensity projections (YX, YZ, and ZX) of fluorescent bead volume imaged using the OPM system. Scale bar: 10 µm. (b) Example XY and ZY views of a single fluorescent bead. Scale bar: 1 µm. (c) Normalized intensity profiles (dots) and corresponding Gaussian fits (curves) extracted along the dashed lines in panel (b) for the example bead. The measured lateral and axial FWHM were 0.46 and 0.67 µm, respectively. (d) Quantification of lateral and axial resolution based on FWHM measurements of 90 individual beads. Data are presented as mean ± standard deviation. All data groups passed the D'Agostino‐Pearson normality test, confirming the Gaussian distribution of measured FWHM values (*p* > significance level α = 0.05).

To experimentally characterise the system resolution, we imaged sub‐diffraction fluorescent beads (TetraSpeck Microspheres, T7279, 100 nm nominal diameter, Thermo Fisher) embedded in 1.2% agarose. A 3D maximum intensity projection of the bead sample is shown in Figure 3a. Since the OPM system is configured to image the upper surface of cells, the tertiary objective O3 was adjusted axially to focus approximately 10 µm above the coverslip. The optimal focus region, defined by the Rayleigh length of the light sheet, was approximately 6 µm in depth. Therefore, only beads located within this range were analysed to ensure accuracy and consistency.

The lateral resolution was then assessed by measuring the FWHM of the intensity profile across the bead image in the XY plane. The axial resolution was determined from the FWHM of the *Z*‐axis intensity profile extracted through the brightest pixel of each bead (see Figure 3b,c for an example).

Averaging across 90 fluorescent beads yielded mean FWHM values of 0.48  ± 0.02 µm (lateral) and 0.67 ± 0.07 µm (axial) (Figure 3d). These experimental measurements are considered acceptable. The moderate deviation from the theoretical estimation is attributed primarily to imaging performed approximately 10 µm above the coverslip, where refractive index mismatch and sample‐induced aberrations can broaden the effective PSF under remote‐refocusing conditions [28, 33]. Notably, the distributions of both lateral and axial resolution measurements passed the D'Agostino‐Pearson test for normality, supporting the robustness of the system's resolution characterisation.

### 3.3 Correlative Volumetric Imaging of Cardiomyocyte Detubulation

Transverse tubules (TTs) are essential membrane structures in cardiomyocytes, formed by deep invaginations of the sarcolemma that extend into the cell interior [34]. TTs enable the rapid and uniform transmission of action potentials throughout the cell and are essential for the excitation–contraction coupling in cardiomyocytes. Structural remodeling of TTs, alongside the loss of membrane z‐grooves where TT openings typically localize, is a hallmark of myocardial infarction and other cardiac pathologies [35]. Importantly, the extent and pattern of TT disruption vary across different disease models and may offer mechanistic insights into disease progression. For example, formamide‐induced detubulation of rat cardiomyocytes occurs via osmotic shock and results in substantial loss of both surface z‐grooves and internal TT structures [36, 37]. In contrast, cationic amphiphilic drugs (CADs), such as imipramine, have been reported to disrupt TTs by dissociating the membrane scaffolding protein BIN1 from PI(4,5)P_2_ [38, 39], although direct 3D visualization of this process has not been previously demonstrated.

**FIGURE 4:**
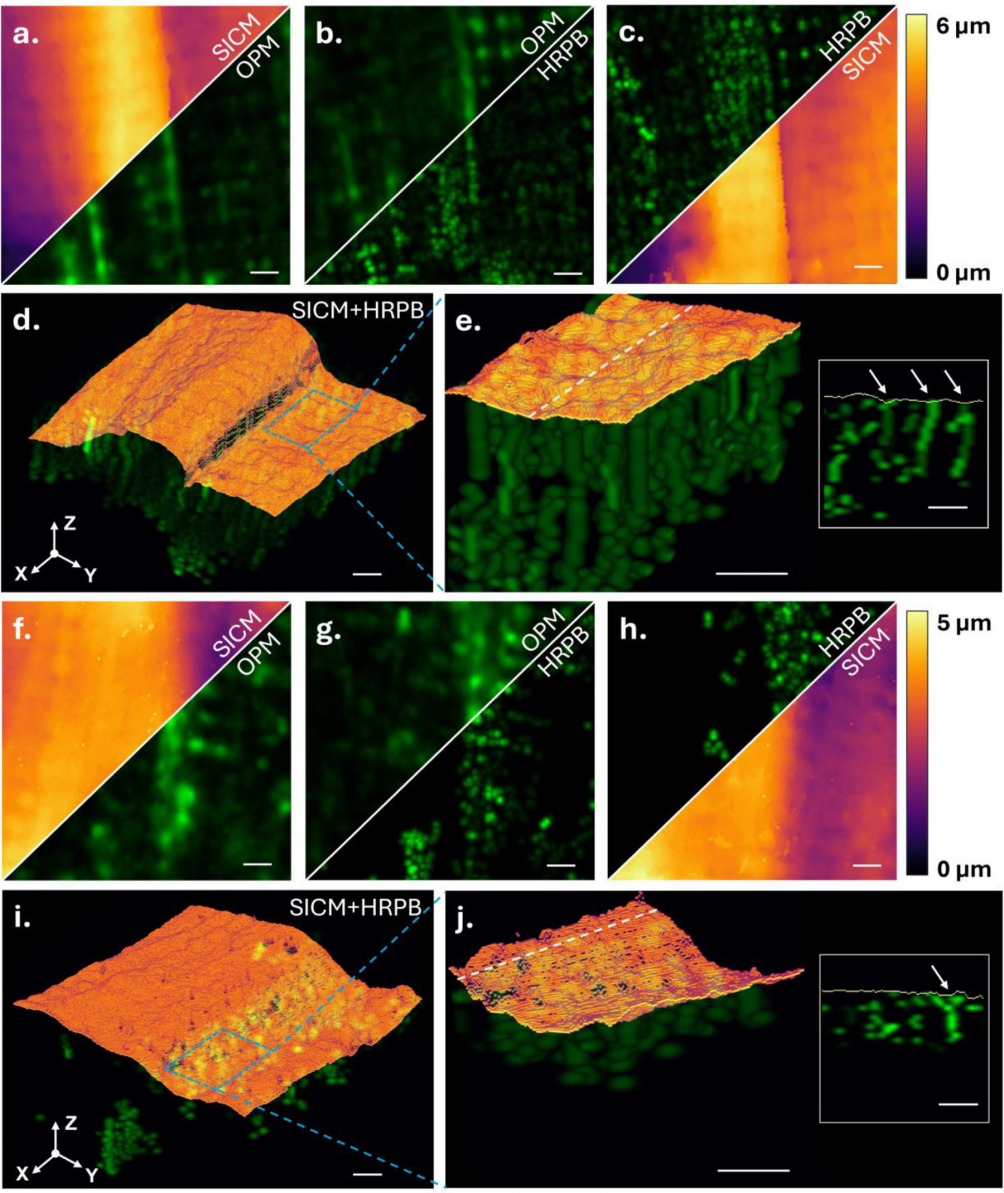
Correlative SICM‐OPM imaging reveals distinct patterns of cardiomyocyte detubulation following imipramine treatment. (a–c) Correlative imaging (20 µm × 20 µm) of a control cardiomyocyte showing (a) SICM topography, (b) OPM fluorescence image, and (c) high‐resolution point‐based (HRPB) reconstruction of Di‐8‐ANEPPS‐labeled transverse‐tubules (TTs). (d) 3D rendering of the SICM+HRPB data from the same cell as (a–c), illustrating intact surface z‐grooves and well‐organized submembrane TT structures. (e) Zoom‐in of the region outlined in (d), highlighting the continuity of subsarcolemmal invaginations. The inset shows a cross‐sectional intensity profile along the white dashed line. The white arrows mark clearly visible TT openings. (f–h) Correlative imaging (20 µm × 20 µm) of a cardiomyocyte treated with 300 µm imipramine showing (f) SICM topography, (g) OPM fluorescence, and (h) HRPB reconstruction. (i) 3D rendering of SICM and HRPB data from the same treated cell, revealing preserved surface topography but disrupted internal TT architecture. (j) Zoom‐in of the region outlined in (i), where the TT openings (white arrows) are markedly reduced compared to the control. Inset: cross‐sectional intensity profile showing loss of invagination continuity. All scale bars: 2 µm. Results shown are representative of ten independent repeats with similar outcomes.

**FIGURE 5:**
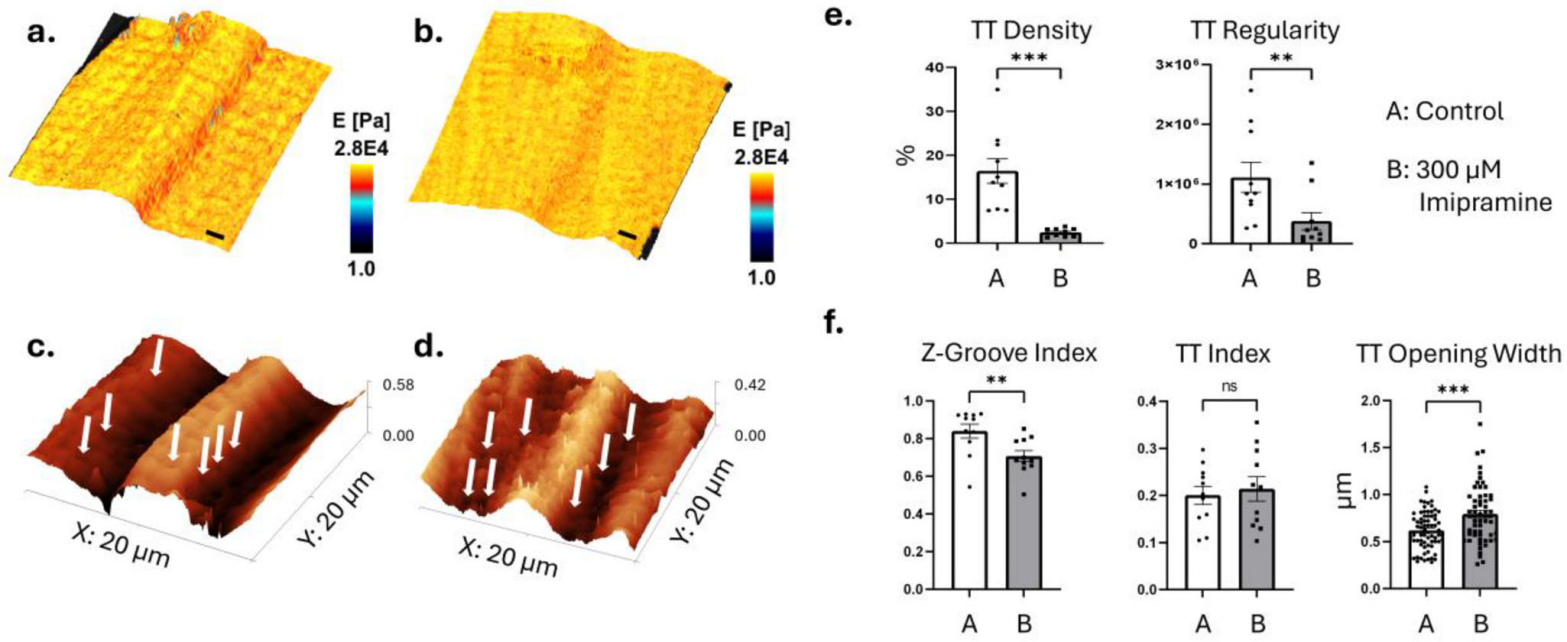
SICM stiffness measurement and quantitative analysis of cardiomyocyte detubulation. (a,b) Corresponding stiffness maps of the control and imipramine‐treated cardiomyocytes are shown in Figure 4. Control mean stiffness 12.064 ± 5.5 kPa (128 × 128 pixels), detubulated mean stiffness 4.975 ± 1.9 kPa (192 × 192 pixels). Scale bars: 2 µm. (c,d) Corresponding 3D SICM surface topography images corrected by second‐order polynomial background subtraction, highlighting TT openings (white arrows). (e) Quantification from OPM images of TT density (control: 16.4% ± 2.8%; imipramine: 2.4% ± 0.3%) and TT regularity (control: 1.11 × 10^6^ ± 2.47 × 10^5^; imipramine: 3.79 × 10^5^ ± 1.43 × 10^5^), corresponding to an 85.1% ± 6.7% reduction (*p *< 0.001) in density and 65.9% ± 14.2% decrease (*p *< 0.01) in regularity. (f) SICM‐derived quantification of z‐groove index (control: 0.84 ± 0.04; imipramine: 0.71 ± 0.03; with a 15.8% ± 5.1% decrease, *p* < 0.01), TT index (control: 0.20 ± 0.02; imipramine: 0.21 ± 0.03; with a 10.7% ± 15.7% decrease, ns), and TT opening width (control: 0.62 ± 0.02 µm; imipramine: 0.79 ± 0.04 µm; with a 27.8% ± 6.3% significant increase, *p*<0.001). Data are presented as mean ± standard error of the mean. Statistical significance: ^**^ *p * <  0.01, ^***^ *p* < 0.001; ns = not significant. *t*‐test was applied in TT Density, TT Index, and TT Opening Width; Mann‐Whitney test was applied in TT Regularity and Z‐Groove Index. In (e): *n* = 10 (10 non‐overlapping 20 µm × 20 µm × 2 µm ROIs within one representative cell). In Z‐Groove Index and TT Index: *n* = 11, where each dot represents one z line. In TT Opening Width: *n* = 69 (control), 58 (detubulation), where each dot represents one TT opening. TT: transverse tubule. Similar results were observed in ten independent experimental repeats.

To address this, we used the SICM‐OPM platform to investigate imipramine‐induced detubulation in adult rat ventricular myocytes (ARVMs) as a proof‐of‐concept demonstration of the system's capability to simultaneously resolve distinct membrane features and intracellular structures. Fluorescence‐labeled TT structures (via Di‐8‐ANEPPS) and high‐resolution surface topography were simultaneously acquired from the same region of interest (Figure 4a; Figure S5). To enhance the visualization of submembrane TT networks, high‐resolution point‐based (HRPB) reconstructions (see Section 2.5) were performed on the OPM fluorescence data (Figure 4b). SICM height maps were corrected by second‐order polynomial background subtraction to eliminate large‐scale curvature and sample tilt. Following background correction, the minimum z value within each image was set to zero to standardise the vertical reference for subsequent quantitative analysis (Figure 5c,d).

Correlative SICM‐OPM imaging showed that untreated ARVMs exhibited intact surface morphology and continuous, well‐defined TT structures (Figure 4a–e). In contrast, ARVMs treated with 300 µm imipramine showed apparent changes of z‐groove index [37] in SICM images (Figures 4f–h and 5f, with a reduction of 15.8% ± 5.1% (*n* = 11, z lines)), suggesting disruption of membrane topography. Notably, we also observed that the total number of TT openings within the analyzed regions differed before and after treatment (69 in control vs. 58 following detubulation). Meanwhile, SICM topography also revealed a significant enlargement of TT openings, with an average width increase of 27.8% ± 6.3% (*n* = 69, TT openings in control group, *n* = 58, TT openings in detubulation group), suggesting disruption of TT opening integrity (see Figure 5f, TT Opening Width). Although the average TT opening width increased after treatment, the TT index for each z line (defined as TT opening width normalized to the corresponding z line length) remained largely unchanged (see Figure 5f, TT Index). Meanwhile, OPM fluorescence imaging indicated a substantial reduction in TT density by 85.1% ± 6.7% (*n* = 10, selected ROIs), and a decrease in TT regularity [40] by 65.9% ± 14.2% (*n* = 10, selected ROIs), as evidenced by fragmented or absent TTs (Figures 4i,j and 5e). Together, these findings suggest that imipramine selectively impairs internal TT invagination and membrane topography in live cardiomyocytes, and alters surface TT opening morphology through width enlargement and partial numerical reduction, rather than complete disruption. In addition, SICM‐based stiffness measurements revealed a decrease in membrane stiffness in cardiomyocytes after the treatment with 300 µm imipramine (Figure 5a,b). These observations were reproducible across ten independent experimental replicates, all of which demonstrated comparable alterations in TT structure.

Importantly, the fluorescence imaging results from our SICM‐OPM platform are highly consistent with previously published confocal microscopy studies. In our prior work [41], ARVMs treated with 300 µm imipramine and imaged by confocal microscopy exhibited a similar reduction in TT density and regularity, consistent with the values reported here. Comparable findings have also been reported by others under equivalent treatment conditions [39]. These concordant findings not only attest to the robustness and accuracy of our SICM‐OPM measurements but also highlight its unique advantage in capturing high‐resolution 3D images of live cardiomyocyte TT networks.

To our knowledge, this is the first demonstration of correlative imaging that simultaneously resolves both the surface topography and intracellular TT architecture within the same live cardiomyocyte. Further studies will leverage this platform to investigate the dynamics and mechanisms underlying cardiomyocyte detubulation in greater depth. Overall, these results highlight the power of SICM‐OPM for visualizing membrane‐associated ultrastructures and detecting subcellular pathological changes with high specificity.

### 3.4 SICM‐OPM‐Guided Delivery of Molecules Into Cells

Observing single‐cell responses to the localized delivery of exogenous cargos offers valuable insights into a range of fundamental biological processes, including antigen recognition, inflammatory signaling, viral entry, and targeted drug delivery. The SICM‐OPM platform facilitates such investigations by leveraging the nanopipette not only as a topographic probe, but also as a highly precise tool for electroporation and molecular delivery [25]. Here, we demonstrate the system's capability for high‐precision, targeted single‐cell injection in both *Coscinodiscus radiatus* diatoms and mammalian neuroblastoma (SH‐SY5Y) cells, highlighting its versatility across phylogenetically and structurally distinct cell types.

**FIGURE 6:**
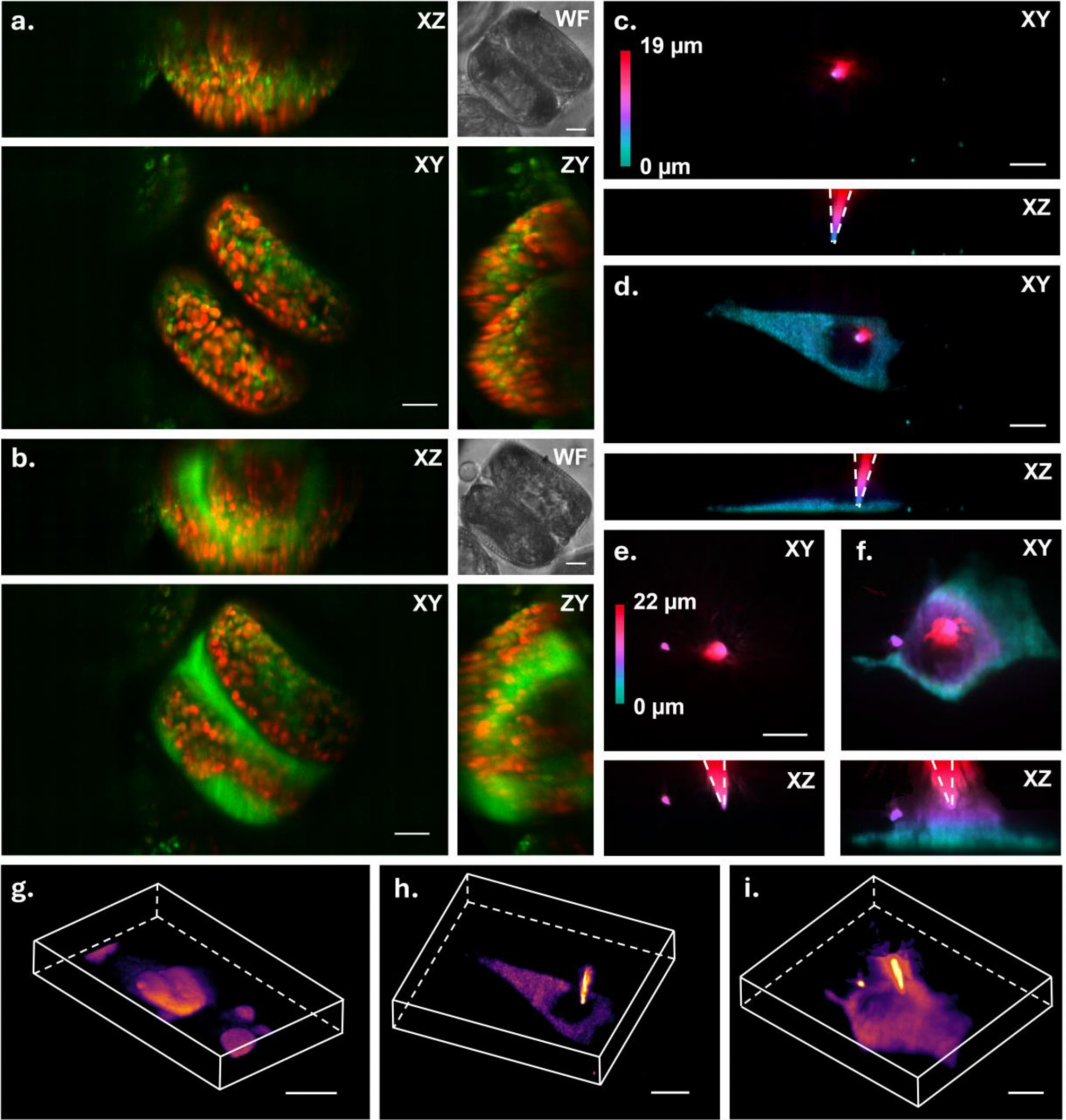
SICM‐OPM enables volumetric visualization of localized single‐cell injection in diatoms and mammalian cells. (a,b) Maximum intensity projections (MIPs) of *Coscinodiscus radiatus* (CCMP312) before (a) and after (b) local electroporation‐based injection of FITC‐Dextran (70 kDa, 46945, Sigma–Aldrich). Each panel includes XY, XZ, and ZY views from OPM, along with a corresponding widefield (WF) image. (c,d) XY and XZ projections of an SH‐SY5Y human neuroblastoma cell injected with fluorescein (5 µm, 46955, Fluka) into the cytoplasm. The SICM nanopipette position is indicated by white dashed lines. (e,f) An SH‐SY5Y cell injected with 70 kDa FITC‐Dextran before (e) and after (f) injection. Dashed lines mark the nanopipette location. (g) 3D rendering of an SH‐SY5Y cell injected with fluorescein into the nucleus. (h,i) 3D views of the cells shown in (d) and (f), respectively, illustrating the spatial distribution of the injected material. All scale bars: 10 µm. All injection patterns were consistently reproduced across five independent experiments.

We first performed targeted injection of FITC‐Dextran (70 kDa, 46945, Sigma–Aldrich) into the girdle region of a *Coscinodiscus radiatus* diatom cell undergoing cytokinesis (Figure 6a,b). While diatom cells are enclosed within two rigid silica‐based frustules, the girdle region remains mechanically softer and more accessible to nanopipette penetration, particularly during cell division before the completion of silica deposition [42]. Fluorescence signals were predominantly localised inside the frustule space between the two closely apposed daughter cells, rather than within the cytoplasm. This region likely corresponds to the developing cleavage plane between two nascent valves, representing a transient and structurally delicate compartment during cytokinesis. The successful delivery of FITC‐Dextran into this intermediate zone highlights the capability of the SICM‐OPM platform in accessing shielded or mechanically constrained subcellular domains and potentially offers a new approach for gene delivery into diatoms.

Next, we applied the SICM‐OPM system for intracellular injection of both low‐ and high‐molecular‐weight fluorescent reagents into SH‐SY5Y cells and monitored their subcellular distribution. Cytoplasmic injection of fluorescein (5 µm, 46955, Fluka) resulted in strong intracellular fluorescence confined within the cytoplasmic volume but not in the nucleus (Figure 6c,d,h). Despite the thin cytoplasmic layer in the cell, electroporation‐based injection was achieved with high precision, highlighting the system's fine positional control. In a separate experiment, fluorescein was selectively injected into the nucleus of another SH‐SY5Y cell (Figure 6g), further demonstrating subcellular targeting precision. Similar localization patterns were observed upon injection of FITC‐Dextran (70 kDa) (Figure 6e,f,i), confirming the system's compatibility with a range of molecular cargos.

**FIGURE 7:**
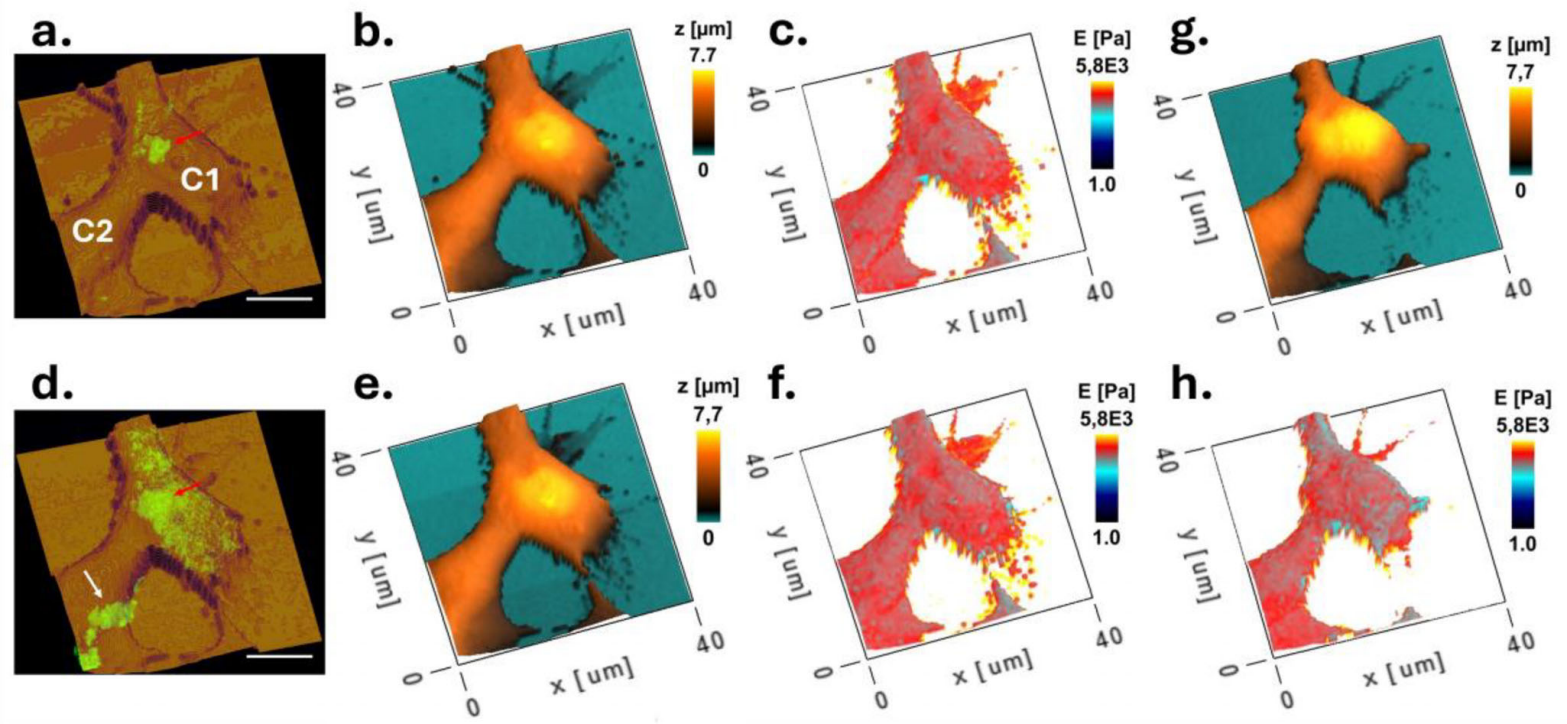
Intracellular delivery of fluorescently labeled α‐synuclein into single cells by localized electroporation via SICM nanopipette and mechanical mapping of injected and non‐injected SH‐SY5Y cells. (a–c) 3D SICM topography and OPM fluorescence overlay image (a) of two adjacent SH‐SY5Y neuroblastoma cells (C1 and C2) before the delivery, and corresponding SICM topography (b) and stiffness (c) maps. (d–f) SICM‐OPM overlay image (d) acquired right after the electroporation showing fluorescently labeled α‐synuclein inside cell C1 cytoplasm and corresponding SICM topography (e) and stiffness (f) maps. (g,h) SICM topography and stiffness map acquired 3 h after the delivery. All scale bars: 10 µm. Results shown are representative of three independent repeats with similar outcomes.

To further demonstrate the multifunctionality of the SICM‐OPM platform, we performed a proof‐of‐concept experiment combining targeted protein delivery with correlative topographical and mechanical mapping. Fluorescently labeled α‐synuclein monomer was injected into an SH‐SY5Y neuroblastoma cell (C1), with an adjacent non‐injected cell (C2) serving as an internal control. Figure 7a shows SH‐SY5Y cells before the delivery. Corresponding SICM topographical and stiffness images are shown in Figure 7b,c. The mean C1 and C2 stiffness before the injection were 463.2 ± 146.7 and 486.6 ± 145.5 Pa.  OPM fluorescence imaging revealed fluorescence in C1 cytoplasm post‐injection (Figure 7d), while C2 remained largely unchanged. Interestingly, a small fluorescent punctum emerged in C2 (highlighted by a white arrow), possibly indicating intercellular diffusion or endocytosis of α‐synuclein. Notably, an autofluorescent feature visible in C1 prior to injection remained stable (indicated by red arrows), confirming cell identification across time points. SICM topographical image acquired right after delivery (Figure 7e) did not reveal any changes to the cell morphology, and the cell's stiffness was 488.0 ± 168.1 Pa for C1 and 491.0 ± 132.8 Pa for C2, indicating that the injection procedure itself is minimally invasive. Example ROI masks are shown in Figure S6a,b. Three hours post‐injection, C1 cell exhibited swelling and rounding (Figure 7g) and softening (363.5 ± 117.2 Pa), but C2 stiffness remained unchanged (488.9 ± 157.2 Pa; Figure 7h). To test whether the change in cell morphology and stiffness was due to the delivery process or more likely caused by α‐synuclein monomers, we injected other SH‐SY5Y cells with 10 000 MW dextran labeled with Alexa Fluor 647 as a neutral molecule (Figure S6c–j). We found that the change in cell stiffness in non‐injected cells over one and a half hours was greater than in the cell injected with dextran, confirming that the delivery by localized electroporation via SICM pipette is minimally invasive. While the influence of α‐synuclein monomers on cell mechanics requires further investigation, this experiment serves as a proof‐of‐concept that SICM‐OPM can deliver defined protein species and track subsequent mechanical responses in individual live cells. These results also demonstrate the platform's capability to deliver protein cargo with subcellular precision and to monitor subsequent mechanical changes in a time‐resolved manner.

Each injection experiment was independently repeated at least three times, with all replicates yielding comparable outcomes, highlighting the reproducibility of the results. Together, these findings present the broad utility of the SICM‐OPM platform in delivering molecules with subcellular precision, while simultaneously capturing correlative topographical and mechanical responses. This integrated approach offers a powerful platform for investigating intracellular transport, cytoskeletal remodelling, and pathophysiological processes, such as protein aggregation in neurodegenerative diseases, with broad potential applications in neuroscience, immunology, and nanomedicine.

### 3.5 High‐Speed Correlative Imaging Using SICM‐OPM

To demonstrate the high‐speed correlative imaging capability of SICM‐OPM platform, we visualised well‐characterized clathrin‐mediated endocytosis in Cos‐7 cells transiently transfected with Clathrin light chain‐GFP (Clc‐GFP) plasmid. The results were reproducible across four independent experimental repeats.

**FIGURE 8:**
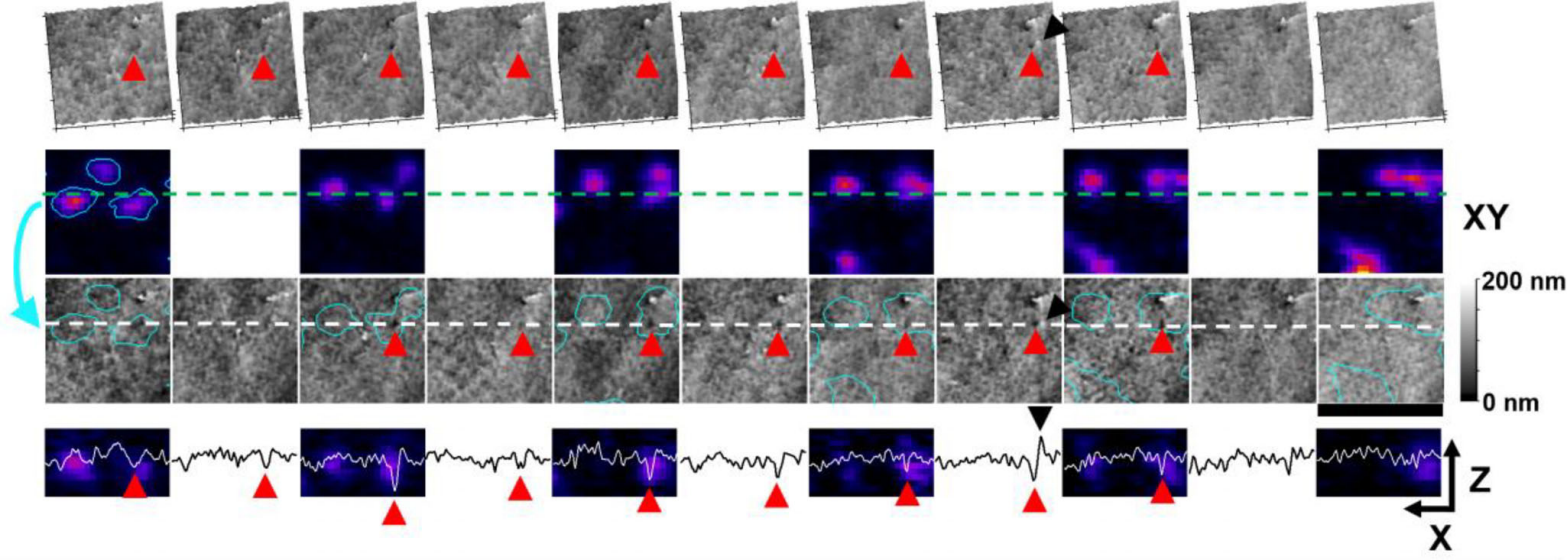
High‐speed SICM‐OPM imaging of clathrin‐mediated endocytosis in Cos‐7 cells. Representative SICM topographical images (top row), red arrow points at indentations corresponding to clathrin‐coated pit. Corresponding maximum intensity projection of OPM images (second row) showing Clc‐GFP fluorescence at the pit site. Overlay of SICM topography and corresponding OPM fluorescence outline (third row). Overlay of SICM topography cross‐section corresponding to dashed line and OPM XZ section views along the dashed line (bottom row). The black arrow points at a characteristic protrusion preceding pit closure. Scale bar: 5 µm. Results shown are representative of four independent repeats with similar outcomes.

A sequence of topographical time‐lapse images reveals a clathrin‐coated pit formation, maturation, and closure that could be seen as the appearance and disappearance of an indentation in the cell plasma membrane approximately 60 nm deep and 250 nm wide, measured as Full Width at Half Maximum (Figure 8, top row, red arrows). Based on Transmission Electron Microscopy data, they typically range from 65 to 135 nm in diameter. The observed difference in pit dimensions is due to the convolution by the geometry of ∼100 nm diameter SICM nanopipette used for imaging. The presence of the clathrin coat is confirmed by spatially and temporally correlated Clc‐GFP fluorescence imaged by OPM (Figure 8, second row). Importantly, the topographical time‐lapse images detect the formation of characteristic cell membrane protrusion (Figure 8, black arrows) preceding the pit closure and disappearance from the cell surface in agreement with previously reported observations [43]. OPM XZ section views show offset of fluorescent spot right after the formation of the protrusion, presumably indicating the departure of the newly formed clathrin‐coated vesicle (Figure 8, bottom row). SICM topographical images were acquired at 64 × 64 pixels with a frame rate of 7 s per frame, representing a fourfold improvement over our previous implementation [13]. OPM fluorescence volume (50 frames) was acquired within 3 s, and synchronized with alternating SICM frames, resulting in fluorescence acquisition every 14 s. Synchronization between the two modalities was implemented at the frame level via hardware triggering. While SICM acquires topographical information through scanning over a 7 s interval and OPM captures each fluorescence volume within a shorter acquisition window, each OPM volume temporally corresponds to the associated SICM frame. This ensures temporal alignment of dynamic events, although intra‐frame acquisition is not pixel‐by‐pixel synchronized. This strategy was implemented to minimize photobleaching of Clc‐GFP while preserving temporal correlation between topographical and fluorescence signals. It is worth noting that the temporal subsampling of fluorescence frames was introduced as a photoprotection strategy rather than a system constraint. For samples with sufficient fluorescence budget, the synchronization of SICM and OPM acquisition for every frame is possible.

## 4 Discussion and Conclusions

The SICM‐OPM system presents a highly versatile platform for volumetric correlative live imaging and precise single‐cell manipulation. To validate its performance across distinct biological contexts, we selected multiple cell models representing different structural and functional challenges. Cardiomyocytes were chosen for their highly organized transverse‐tubule networks with dimensions close to the diffraction limit, providing a rigorous demonstration of correlative surface‐volume imaging. Diatoms, possessing rigid silica cell walls, served as a model for targeted intracellular delivery in mechanically constrained unicellular organisms. Mammalian neuroblastoma cells enabled evaluation of protein delivery combined with time‐resolved mechanical mapping, while Cos‐7 cells expressing Clc‐GFP provided a well‐characterized dynamic system to assess high‐speed spatiotemporal imaging of nanoscale membrane invaginations. These studies collectively rely on the dual functionality of the SICM nanopipette, which can switch seamlessly between high‐resolution topographic mapping and targeted intracellular delivery, thereby expanding the system's applicability to a broad range of biological investigations. Together, these models demonstrate the robustness, adaptability, and multifunctionality of the SICM‐OPM platform across structurally diverse biological systems.

SICM has been combined with confocal microscopy in previous studies to enable simultaneous topographical and molecular fluorescence visualization [12, 14]. However, those implementations with SSCM and with CLSM are either limited to surface fluorescence acquisition or rely on sequential optical sectioning through mechanical axial scanning to reconstruct volumetric fluorescence information. As a result, these architectures limit temporal resolution and increase photobleaching during live‐cell imaging. Other strategies have also explored combining SICM with fluorescence imaging that reconstructs volumetric information through statistical analysis of temporal signal fluctuations [16], but these often require long acquisition times and extensive post‐processing, limiting their suitability for real‐time applications. In contrast, SICM‐OPM addresses these challenges through key architectural innovations. First, the sample remains stationary while the SICM nanopipette is scanned in 3D using a combination of tip‐tilt and single‐axis piezoelectric micromanipulators. Second, the integration with OPM allows for fast volumetric fluorescence imaging without the need for z‐stack acquisition over volumes comparable to those scanned by SICM, thus enabling true volumetric correlation within seconds.

The ability of SICM‐OPM to perform high‐speed, high‐resolution, and minimally invasive correlative imaging provides a substantial advantage for studying live cellular dynamics in 3D. The OPM architecture presented here is also compatible with super‐resolution imaging techniques. In principle, single‐molecule localization microscopy (SMLM) can be implemented within an OPM geometry. However, SMLM typically requires the acquisition of thousands of frames under high excitation intensities, which limits its application in long‐term live‐cell imaging. Consequently, it is more commonly employed for imaging fixed samples [44]. More suitable strategies for super‐resolution live‐cell imaging include super‐resolution optical fluctuation imaging (SOFI) and structured illumination microscopy (SIM). Among these, SOFI holds particular advantages, as it primarily relies on computational methods to enhance resolution. It can achieve approximately a twofold improvement in spatial resolution without requiring significant modifications to the current hardware architecture [16]. SIM could also be integrated with OPM to achieve super‐resolution imaging for live cells, but this would require patterned illumination and additional optical components in the excitation path, thereby increasing the overall system complexity [45].

In addition, although the pixel frequency of high‐speed SICM reported here is moderate, 584 Hz, which is approximately ten times lower than the fastest high‐speed SICM reported to date to our knowledge [46], it is on par with other high‐speed SICM microscopes [47]. At the same time, the topographical resolution of our high‐speed SICM topographical images is sufficiently high to clearly resolve 100‐nm structures in the cell plasma membranes. It is also important to note that to achieve high‐speed volumetric fluorescence imaging, the sample must be kept still during image acquisition. Therefore, we chose to construct a 3D pipette piezo scanner that resulted in a lower overall resonant frequency due to piezo actuator stacking. The SICM architecture presented here provides virtually unrestricted access to samples and enables the use of standard laboratory petri dishes and multi‐well plates. One promising application is the study of mechanosensory neurons [48], which convert mechanical deformation into electrical or biochemical signals. SICM enables non‐invasive, high‐resolution probing of local membrane mechanics or specialised nerve terminals, while OPM can simultaneously capture intracellular calcium transients or neuronal firing using calcium indicators, thereby investigating stimulus‐response coupling in mechanotransduction [49]. Another potential application lies in the investigation of ferroptosis, a non‐canonical form of programmed cell death driven by membrane lipid peroxidation [50]. While the primary biochemical changes occur intracellularly, such as iron overload, reactive oxygen species (ROS) accumulation, and mitochondrial dysfunction, these processes also lead to secondary alterations in the plasma membrane's nanomechanical properties and integrity. SICM can sensitively detect such nanoscale disruptions on the cell surface, including changes in stiffness, which reflect ferroptotic damage. Concurrently, OPM can image the intracellular biochemical events using appropriate fluorescent reporters [51], enabling a comprehensive, correlative view of the spatiotemporal progression of ferroptosis. The targeted delivery capability of the SICM‐OPM system further extends its utility to functional studies of exogenous gene and protein expression. This is particularly promising in the context of genetic engineering of diatoms, where reliable and minimally invasive delivery methods are much required [52]. In addition, plasmids can be precisely injected by the nanopipette into the nucleus or cytoplasm to induce gene expression, while cellular dynamics are monitored in real time by OPM. Similarly, direct delivery of functional proteins, such as transcription factors, signaling molecules, cytokines, or even protein aggregates, enables investigation of their immediate effects on cell phenotype and signaling pathways. These capabilities can be readily integrated with downstream single‐cell omics technologies, such as single‐cell RNA‐seq or ChIP‐seq, to correlate functional responses with transcriptional or epigenetic changes, providing a comprehensive, multi‐layered view of cell state. This feature opens new possibilities for studying compartment‐specific signaling, organelle‐specific drug responses, or spatially restricted biomolecular interactions. Such targeted perturbations are valuable for drug target validation, enabling direct investigation of target localization, function, and the phenotypic consequences of local manipulation.

Furthermore, several future enhancements could significantly extend the system's versatility. First, optimizing the light‐sheet‐based OPM for a larger imaging volume would enable comprehensive volumetric imaging of tissue and organoids. Second, by performing motorized stage translation between sequential volumetric imaging sessions, the system could achieve high‐resolution imaging across extended or large‐format biological specimens. Thirdly, the integration of an environmental chamber or live‐cell incubator would support long‐term physiological experiments under tightly controlled conditions, crucial for studies on development, regeneration, or pharmacological response. On the SICM side, expanding the lateral scanning range and improving the responsiveness of piezo actuators would allow for faster, large‐area imaging without compromising resolution, paving the way for higher‐throughput investigations.

Overall, the SICM‐OPM platform represents a powerful tool for live‐cell investigations, offering unique capabilities for membrane morphology characterization, targeted delivery, and functional imaging in a label‐compatible, minimally invasive manner. These integrated capabilities are expected to empower fundamental discoveries across diverse biological fields, including neurodegeneration, inflammation, immune response, and cancer biology.

## Disclosures

A.S. and Y.K. are shareholders in ICAPPIC, Ltd., a company commercializing nanopipette‐based instrumentation.

## Conflicts of Interest

The authors declare no conflicts of interest.

## Supporting information




**Supporting File**: advs75222‐sup‐0001‐SuppMat.pdf.

## Data Availability

The data that support the findings of this study are openly available in Zenodo at Ref. [53].
