# Supplementary material for: A Correlative SICM‐OPM Platform for Surface and Volumetric Imaging in Live Cells
Source: Adv Sci (Weinh). 2026 Apr 9:e75222. Online ahead of print. doi: 10.1002/advs.75222 (PMC13334630; doi:10.1002/advs.75222)
Supplement: Supplementary file 1 — Supporting File: advs75222‐sup‐0001‐SuppMat.pdf. [file ADVS-9999-e75222-s001.pdf]

A Correlative SICM-OPM Platform for Surface and Volumetric Imaging in Live Cells

Wenzhi Hong[1✉*], Ziwei Zhang[2*], Ao Li[3], Ting Sun[3], Yunzhao Wu[2], Devkee M. Vadukul[4], Dylan Jones[6], Bing Li[2], Fengjie Liu[6], Francesco A. Aprile[4,5], Yuri Korchev[1], Julia Gorelik[3], David Klenerman[2] and Andrew Shevchuk[1✉]

[1] Faculty of Medicine, Imperial College London, London, UK

[2] Department of Chemistry, University of Cambridge, Cambridge, UK

[3] National Heart and Lung Institute, ICTEM, Imperial College London, London, UK

[4] Department of Chemistry, Molecular Sciences Research Hub, Imperial College London, London, UK

[5] Institute of Chemical Biology, Molecular Sciences Research Hub, Imperial College London, London, UK

[6] Grantham Institute-Climate Change and the Environment, Department of Life Sciences, Imperial College London, London, UK

✉For correspondence: wenzhi.hong19@imperial.ac.uk; a.shevchuk@imperial.ac.uk
*** These authors contributed equally to this work.**

Supplementary Figures

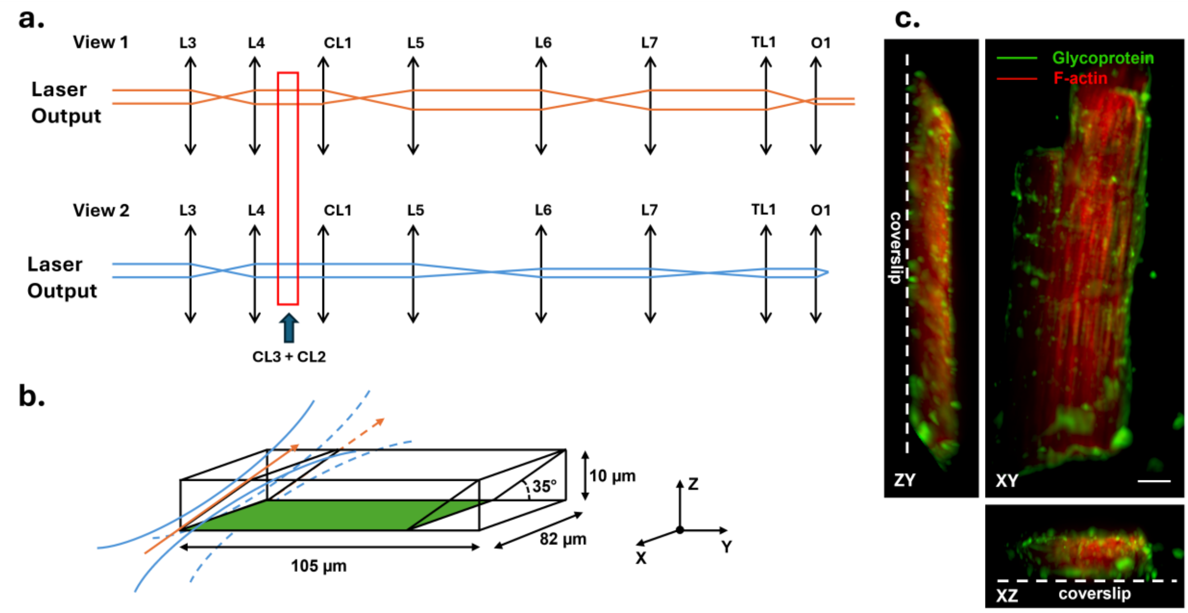

Fig. S1. Light sheet shaping and imaging volume of the OPM system. (a) Optical ray diagram illustrating the laser beam paths for two orthogonal illumination views (View 1: orange; View 2: blue). After passing through a cylindrical lens (CL1), the beam is focused in one direction while remaining collimated in the orthogonal direction. The subsequent lens elements maintain this anisotropy, forming a thin light sheet at the sample plane. The boxed region (CL2 + CL3) indicates the cylindrical lens pair used to expand the beam laterally. (b) Schematic of the OPM light-sheet scanning volume. The light sheet enters the sample at a 35° angle relative to the coverslip and produces a ~105 µm×45 µm×10 µm imaging volume with high optical sectioning quality. (c) Example volumetric image of a cardiomyocyte stained for F-actin (red) and glycoproteins (green), showing the 3D coverage of the system relative to the coverslip. Scale bar: 10 µm. Cells in (c) were co-stained with WGA-Alexa Fluor™ 488 (10 µg/mL) and CellMask™ Deep Red (1 µM)

in high potassium buffer for 30 min at room temperature in the dark. After two washes, samples were imaged immediately using light-sheet microscopy.

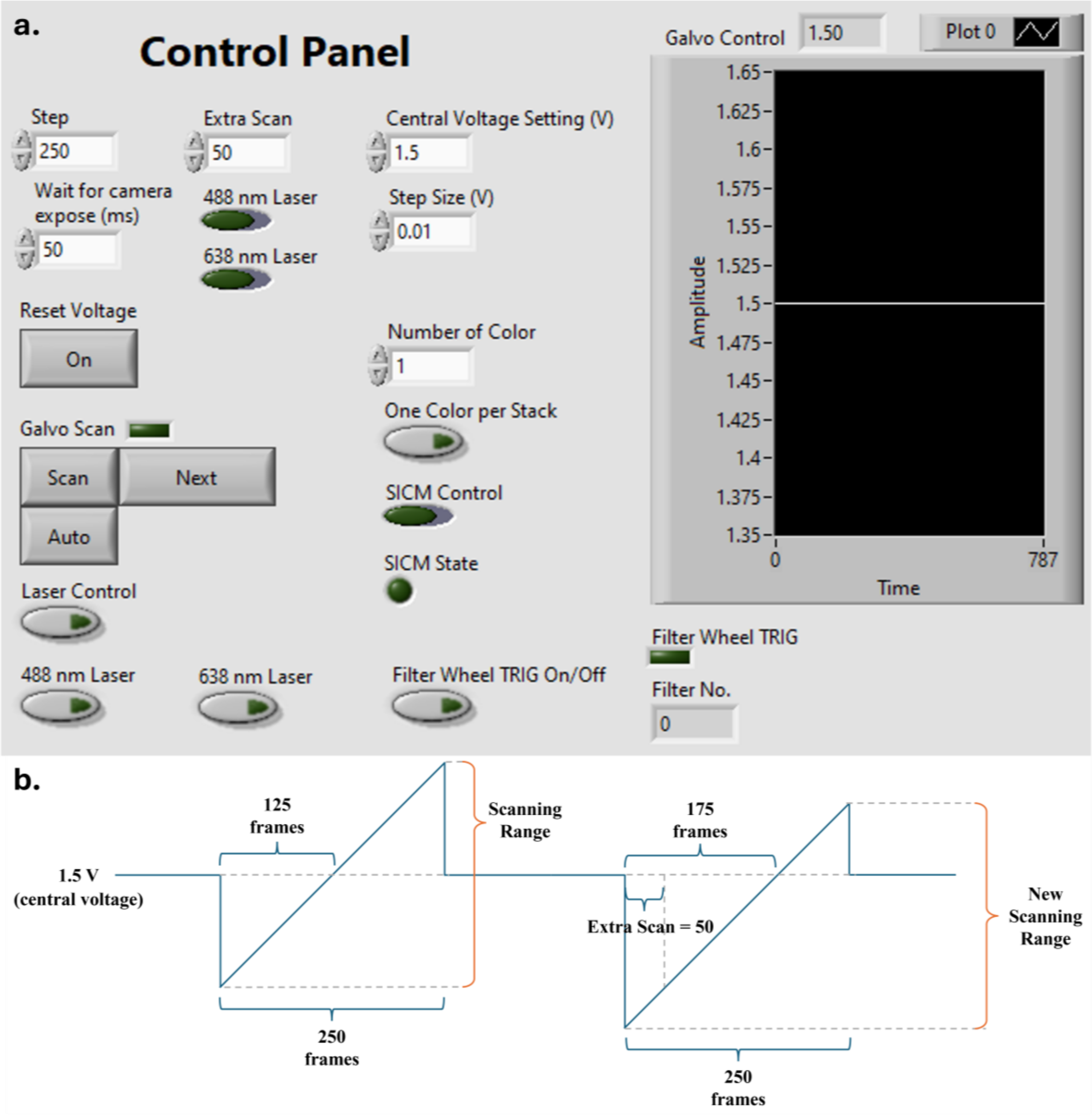

Fig. S2. Galvo scanning control interface and scan voltage profile. (a) Custom LabVIEW-based user interface for controlling the galvo mirror during light-sheet scanning. Adjustable parameters include step count, extra scan offset, central voltage, voltage step size, and camera exposure delay. The interface also allows toggling of laser lines (488 nm, 638 nm), filter wheel triggering, and scanning modes. Real-time voltage output is visualised in the "Galvo Control" plot window during acquisition. The "SICM Control" enables synchronised OPM imaging with SICM scanning. When activated, the camera waits for a rising-edge trigger signal from the SICM controller to initiate image acquisition. (b) Schematic of the galvo mirror voltage profile over time. A typical scan consists of 250 steps, with 125 frames collected per direction around a central voltage (e.g., 1.5 V). The "Extra Scan" parameter (e.g., 50) shifts the scanning origin, generating an offset in the scanning range to accommodate asymmetric sample positioning or increase imaging coverage. This flexible scanning profile enables precise alignment and adjustment of the imaged volume.

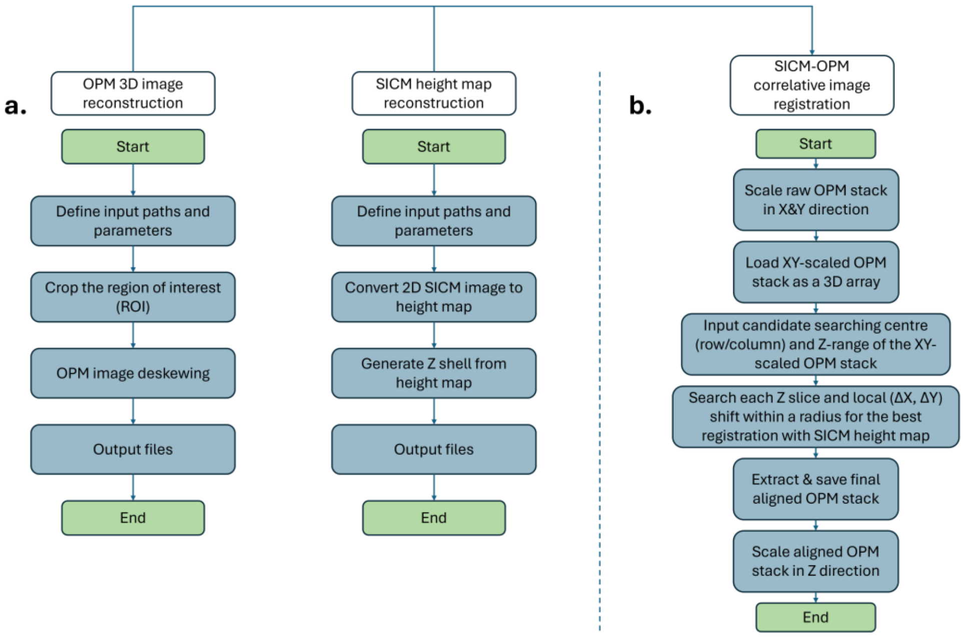

Fig. S3. Flowcharts of (a) OPM 3D image reconstruction, SICM height map reconstruction and (b) SICM-OPM correlative image registration.

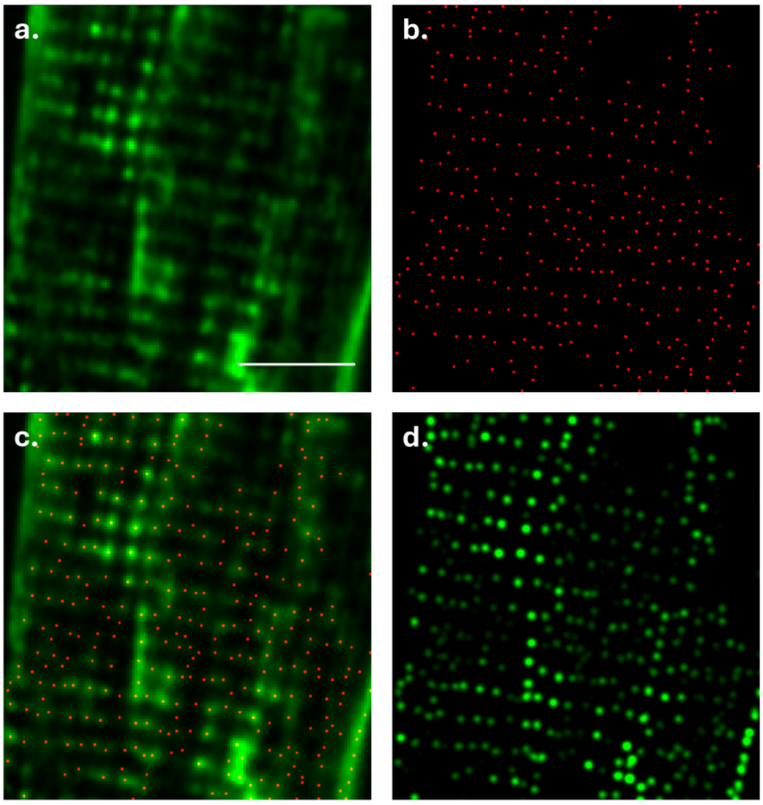

Fig. S4. Workflow for generating high-resolution point-based reconstructions from raw fluorescence data. (a) Raw OPM fluorescence image (a single frame) showing periodic subcellular structures. (b) Localisation map of detected features based on Laplacian of Gaussian (LoG) filtering and thresholding; red dots indicate identified peak positions. (c) Overlay of localisations (red) onto the original fluorescence image.

(d) High-resolution point-based reconstruction, where each localisation is rendered with a Gaussian kernel onto a high-resolution grid, revealing improved structural clarity. Scale bar: 10 µm.

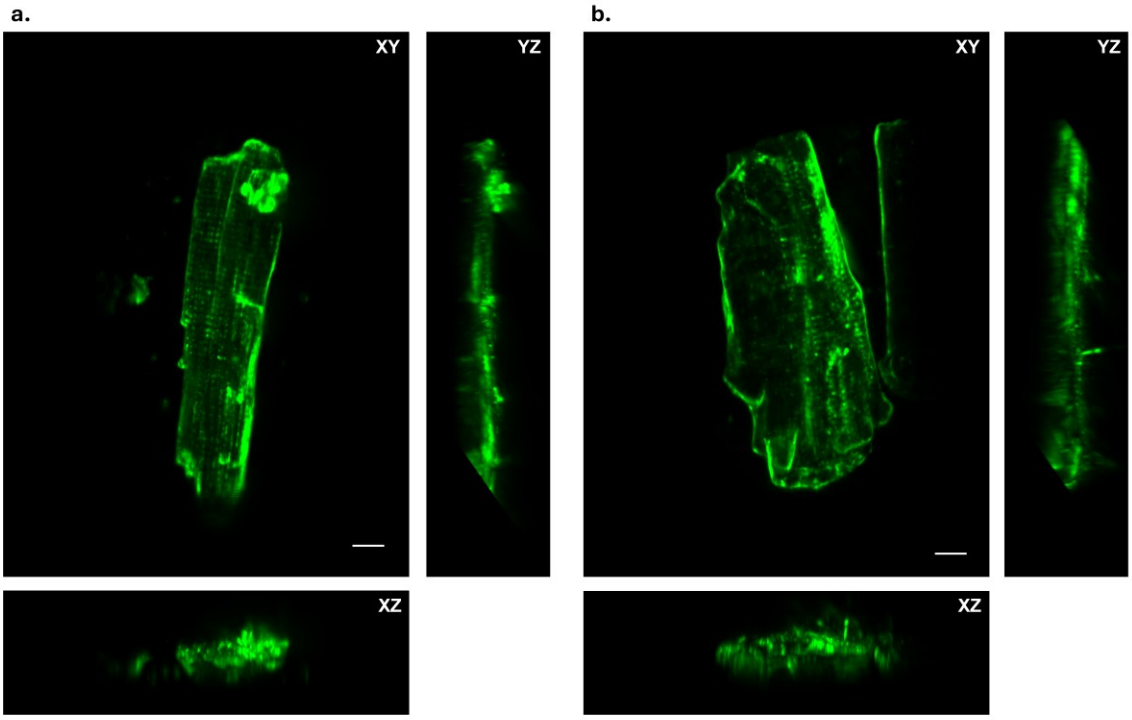

Fig. S5. Full-field OPM fluorescence images of control and detubulated cardiomyocytes. (a) Maximum intensity projections (MIPs) of a control adult rat ventricular myocyte (ARVM), displaying well-organised transverse (T)-tubule structures. (b) Corresponding views of an ARVM following imipramine treatment, showing reduced T-tubule signal intensity and disrupted structural continuity, consistent with detubulation. Each panel presents XY, YZ, and XZ MIP views of the same cell. Scale bars: 10 µm.

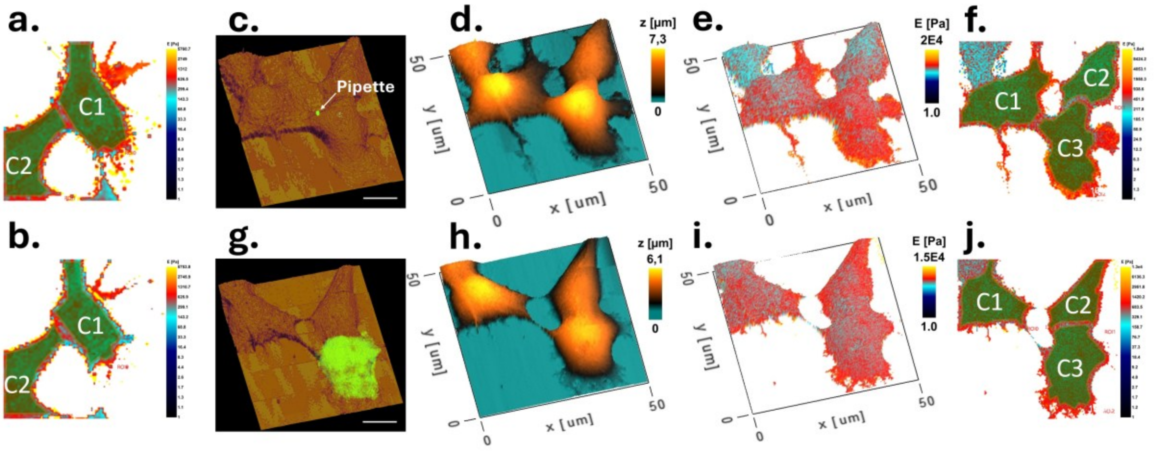

Fig. S6. Quantitative comparison of stiffness changes in SH-SY5Y cells injected and non-injected with α-synuclein and 10,000MW dextran Alexa fluor 647. (a, b) SICM Young's modulus (E) maps showing ROIs corresponding to cells C1 and C2 in Fig. 5 before (a) and 3 hours post-injection (b). (c-f) 3D SICM topography and OPM fluorescence overlay image (c) of three adjacent SH-SY5Y cells (C1, C2 and C3) before electroporation of fluorescently labelled dextran showing the position of SICM pipette above the cell C3 visible as fluorescence spot, corresponding SICM topography (d), stiffness (e) images and ROIs map (f). Cells stiffness C1: 531±266.1 Pa; C2: 405.1± 186.1 Pa; C3: 659.9± 314.8 Pa. (g - j) SICM-OPM

overlay image (d) acquired one and a half hour after the electroporation showing fluorescently labelled dextran inside cell C3 cytoplasm, corresponding SICM topography (h), stiffness (i) and ROIs map (j). Cells stiffness C1: 652.4±293.9 Pa; C2: 735.5±280.4 Pa; C3: 606.5±188.3 Pa. All scale bars: 10 µm. Results shown are representative of three independent repeats with similar outcomes.
